# Linking Soil Properties to Grain Quality: Individual and Combined Effects of Rice Husk Biochar and Potassium Humate in Paddy Rice Systems

**DOI:** 10.3390/plants15203086

**Published:** 2026-10-09

**Authors:** Thidarat Rupngam, Patchimaporn Udomkun, Joachim Müller, Thirasant Boonupara, Puangrat Kaewlom

**Affiliations:** 1 Department of Environmental Engineering, Faculty of Engineering, Chiang Mai University, Chiang Mai 50200, Thailand; thidarat.rupngam@cmu.ac.th; 2 Office of Research Administration, Chiang Mai University, Chiang Mai 50200, Thailand; 3 Institute of Agricultural Engineering, Tropics and Subtropics Group, University of Hohenheim, 70599 Stuttgart, Germany; joachim.mueller@uni-hohenheim.de; 4 Living Soil Co., Ltd., Chiang Mai 50100, Thailand; jarmore001@gmail.com

**Keywords:** soil properties, plant nutrient uptake, rice grain characteristics, rice grain nutrition, rice grain bioactive compounds

## Abstract

The integrated effects of biochar and humate on soil nutrient status, shoot nutrient accumulation, and grain composition and quality remain underexplored. This field study evaluated four treatments—control, rice husk biochar (RHB; 15 t/ha), potassium humate (KH; 6.3 kg/ha), and RHB + KH—on soil properties, shoot nutrient accumulation, grain nutrient composition, yield, and grain quality in a premium Thai rice production system. Compared with the control, RHB significantly increased soil pH and shoot accumulation of nitrogen (N; +93%), phosphorus (P; +321%), and potassium (K; +75%) and resulted in a greater grain P concentration and milled grain yield (+56%). RHB also increased volumetric expansion (+42%), with a significant RHB main effect detected in the factorial analysis. KH significantly increased soil total N (+11%) and ammonium (+19%), shoot N (+79%) and P (+363%) accumulation, and grain N concentration relative to the control, while also increasing milled grain yield (+144%) and grain protein content. KH also produced numerical increases in volumetric expansion (+21%) and grain elongation (+4%), although these responses were not significant in the factorial analysis. The RHB + KH treatment significantly increased soil total N (+18%), shoot N (+70%) and P (+423%) accumulation, and the grain K concentration relative to the control and produced treatment-specific changes in the amino acid profile. The amendment treatments generally decreased residual soil nitrate and available P concentrations, although the factorial response of nitrate included a significant RHB effect and RHB × KH interaction but no significant KH main effect. Grain carbohydrate content decreased under the amendment treatments, whereas TPC showed a treatment-specific response characterized by a significant KH effect and RHB × KH interaction. Principal component analysis revealed associations among soil properties, shoot nutrient accumulation, grain nutrient composition, yield, and grain quality attributes. These findings demonstrate distinct effects of RHB and KH, individually and in combination, on soil properties, shoot nutrient accumulation, and grain composition and quality. Overall, the amendments showed potential for modifying soil nutrient dynamics and shoot nutrient accumulation while altering selected grain nutritional and market-relevant quality attributes, although responses varied among individual parameters.

## 1. Introduction

Rice (*Oryza sativa* L.) is one of the most important staple crops worldwide, with global production averaging approximately 760 million tonnes annually over the past decade [1]. Thailand is among the world’s leading rice exporters, producing around 20 million tonnes annually during the last five years [2] and exporting approximately 8 million tonnes per year [3]. Thai rice, particularly the premium Hom Mali (Khao Dawk Mali) variety, accounts for about 6 million tonnes of annual production and holds a strong position in international markets due to its distinctive aroma, soft texture, and high consumer demand. Beyond its global reputation, rice production also supports millions of farming households and plays a crucial role in Thailand’s agricultural economy [4]. Increasing global competition has heightened the demand for high-quality rice grains, underscoring the importance of soil and nutrient management strategies that enhance both productivity and grain quality [5]. However, intensive rice cultivation often leads to nutrient depletion, soil acidification, and the loss of soil organic matter (OM), which threaten long-term soil fertility and grain quality.

Among organic soil amendments, biochar has gained considerable attention due to its capacity to improve nutrient retention, water-holding capacity, and microbial activity [6]. In rice production systems, rice husk biochar (RHB) is particularly attractive because rice husks are widely available as an agricultural by-product and contain high levels of silica [7]. Previous studies have shown that RHB can increase soil pH, cation exchange capacity (CEC), and nutrient availability, thereby enhancing rice growth and yield [8,9]. In addition to yield improvements, biochar has been reported to influence several grain quality characteristics, leading to higher brown rice, milled rice, and head rice recovery rates, reduced grain chalkiness, increased grain length-to-width ratios, and increased market value [10,11]. Biochar may also improve the starch viscosity and eating quality of rice [12]. Nevertheless, the effects of biochar can vary depending on application rate, environmental conditions, and feedstock type. Recent evidence further indicates that the effects of RHB on rice yield and grain quality can interact with nutrient and water management, highlighting the importance of evaluating biochar within specific production systems [13]. Nevertheless, the effects of biochar can vary depending on application rate, environmental conditions, and feedstock type. For example, reductions in protein and amylose contents have been observed in saline–sodic soils [14], while variations in grain appearance traits have been reported across growing seasons [15].

Potassium humate (KH), a salt derived from humic substances, represents another promising amendment for improving soil fertility and crop performance. KH enhances nutrient solubility, promotes soil aggregation and water-holding capacity, and stimulates plant physiological processes through hormone-like activity [16,17]. It can promote root development, increase chlorophyll content, enhance stress tolerance, and function as a chelating agent that improves micronutrient availability [18,19]. In rice cultivation, humic substances have been reported to improve grain processing quality, including hulling, milling, and head rice percentages, as well as kernel elongation, amylose and protein contents, lipid and ash concentrations, and grain phosphorus (P) and potassium (K) levels [20]. In addition, KH application has been shown to increase soil electrical conductivity (EC), CEC, available nitrogen (N), P and K, soil organic carbon, dehydrogenase activity, and rice yield [21].

Although numerous studies have investigated the individual effects of biochar or humic substances on soil fertility and crop productivity, most have primarily focused on changes in soil physicochemical properties, plant growth, plant nutrient status, or grain yield [22]. Only a limited number of studies have explored their combined application [23,24,25], and these have largely evaluated agronomic performance rather than integrated responses across soil properties, plant nutrient status, and grain composition and quality. Consequently, the relationships among changes in soil nutrient availability, shoot nutrient accumulation, and grain nutrient composition and quality remain insufficiently understood. Understanding these linkages is important because improvements in soil fertility do not necessarily correspond to greater nutrient accumulation in plant biomass or changes in grain nutritional composition and quality. Furthermore, little information is available on how the co-application of biochar and humic substances affects these interconnected soil, plant, and grain responses. Unlike previous studies that have evaluated individual system components separately, the present study integrates measurements of soil chemical properties, shoot nutrient accumulation, and grain nutrient composition and quality to provide a comprehensive assessment of responses to the individual and combined application of RHB and KH. This integrated approach enables the relationships among soil nutrient status, shoot nutrient accumulation, and grain composition and quality to be evaluated within the same field experiment.

Therefore, this study aimed to evaluate the individual and combined effects of RHB and KH on soil nutrient dynamics, shoot nutrient accumulation, yield, and grain composition and quality in a flooded rice production system. Specifically, the study examined their effects on (i) soil chemical properties and nutrient dynamics; (ii) shoot N, P, and K accumulation and yield performance; and (iii) rice grain nutrient composition and quality attributes, including appearance characteristics, cooking properties, and nutritional and bioactive compound compositions. It was hypothesized that (i) the individual application of RHB or KH would modify soil chemical properties and nutrient availability, thereby affecting shoot nutrient accumulation, yield, and grain quality, and (ii) RHB and KH would interact in their effects on soil nutrient status, shoot nutrient accumulation, yield, and grain composition and quality, such that the response to one amendment would depend on the presence of the other.

## 2. Results and Discussion

### 2.1. Soil Chemical Properties and Nutrient Transformations

#### 2.1.1. Soil pH and EC

**Figure 1:**
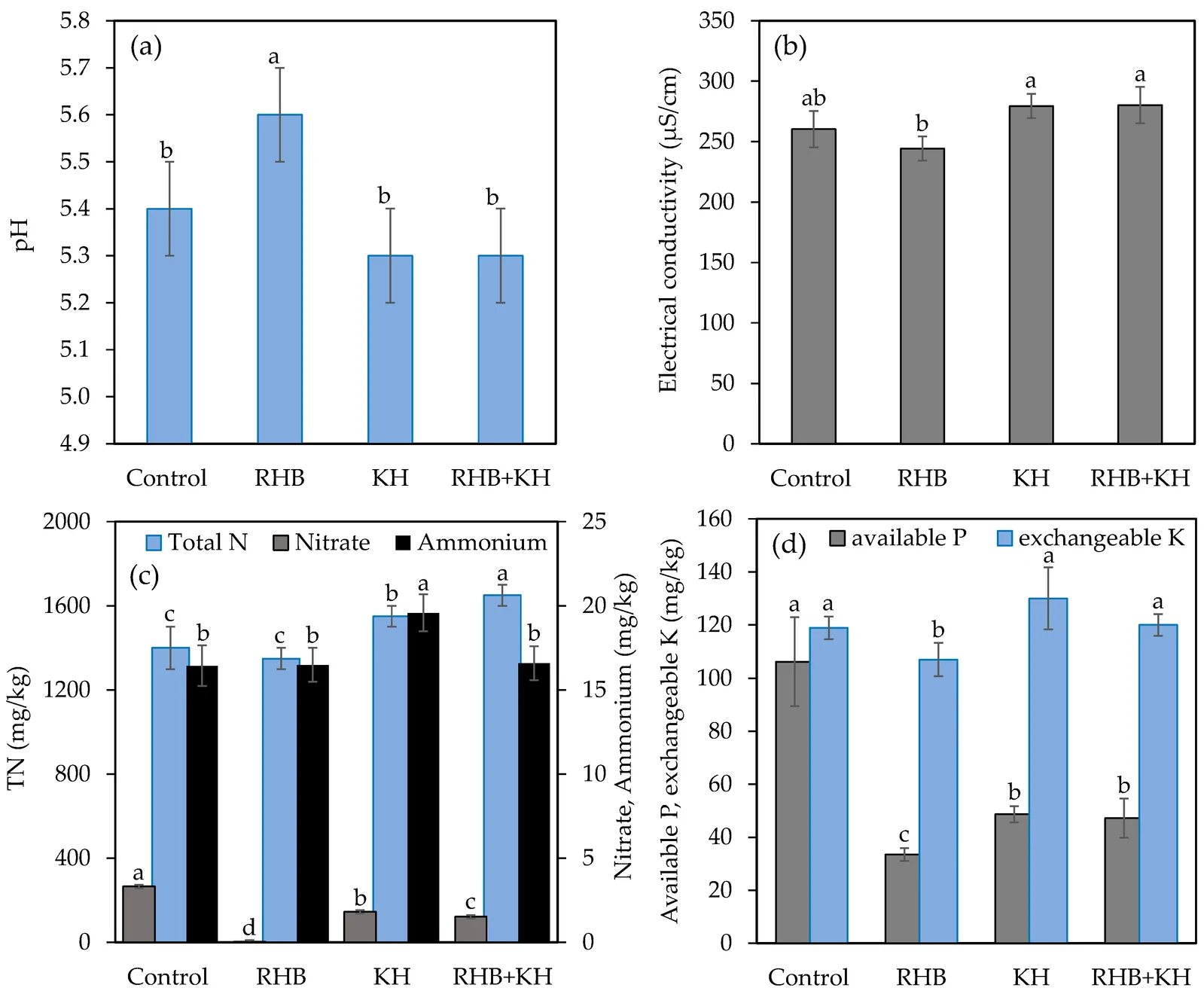
Soil properties at post-harvest, including (**a**) pH, (**b**) electrical conductivity (EC), (**c**) total nitrogen (TN; left *y*-axis) and nitrate (NO_3_^−^) and ammonium (NH_4_^+^) concentrations (right *y*-axis), and (**d**) available phosphorus (P) and exchangeable potassium (K). Values are means ± standard deviation (SD; *n* = 4). Different lowercase letters indicate significant differences among treatments (*p* < 0.05).

Post-harvest soil pH differed among treatments (Figure 1a). Factorial ANOVA indicated significant effects of RHB, KH, and their interaction (RHB × KH; all *p* < 0.001; Table A1). Application of RHB increased soil pH from 5.4 in the control to 5.6, representing a 3.7% increase (Figure 1a). This response is consistent with the well-established liming effect of biochar. RHB contains alkaline ash components, including carbonates, oxides, hydroxides, and base cations (e.g., Ca^2+^, Mg^2+^, and K^+^), which consume protons, displace exchangeable H^+^ and Al^3+^ from soil exchange sites, and promote the precipitation of Al compounds. These processes reduce soil acidity and increase soil pH, particularly in acidic soils [26,27].

In contrast, soil pH under the KH treatment remained statistically similar to that of the control despite the relatively high pH of KH itself (approximately 10.8). Although the mean pH was numerically lower under KH (5.3) than under the control (5.4), this difference was not statistically significant (Figure 1a) and therefore should not be interpreted as KH-induced soil acidification. The absence of a measurable liming effect was likely related to the relatively low KH application rate (6.3 kg/ha), which supplied only a small quantity of alkaline material relative to the soil mass and its inherent buffering capacity. Consequently, the intrinsic pH of the concentrated KH product does not necessarily predict its effect on bulk soil pH after field application. Humic substances also contain carboxyl and phenolic functional groups that participate in proton exchange, metal complexation, and soil buffering reactions [28], which may further moderate changes in soil pH.

In addition to these chemical processes, humic substances have been reported to stimulate microbial activity, root growth, and rhizosphere metabolism, potentially increasing the release of low-molecular-weight organic acids such as citric and malic acids [29,30]. Such processes may contribute to pH regulation within the rhizosphere and illustrate why the response of soil pH to humic substances can be more complex than would be predicted from the pH of the amendment alone. However, because microbial activity, rhizosphere chemistry, and organic acid production were not evaluated in the present study, these processes should be considered possible contributing mechanisms rather than direct explanations for the observed pH response.

Similarly, soil pH under RHB + KH did not differ significantly from the control or KH treatment, whereas RHB alone significantly increased soil pH (Figure 1a). The addition of KH to RHB did not produce a further increase in soil pH, and this non-additive response is consistent with the significant RHB × KH interaction detected by factorial ANOVA (*p* < 0.001; Table A1). Although the mechanism underlying this response was not directly evaluated, interactions among alkaline minerals, exchangeable cations, and oxygen-containing functional groups of humic substances may influence soil buffering and pH regulation when biochar and humic substances are co-applied [31,32].

Soil EC also differed among treatments (Figure 1b), with factorial ANOVA indicating significant effects of RHB, KH, and their interaction (all *p* < 0.001; Table A1). The KH and RHB + KH treatments exhibited the highest EC values (279.4–280.2 µS/cm), representing increases of 7.3–7.6% relative to the control (260.3 µS/cm). In contrast, EC decreased to 244.3 µS/cm under RHB alone, representing a 6.1% reduction relative to the control. Pairwise comparisons showed that EC was significantly lower under RHB than under KH and RHB + KH, whereas KH and RHB + KH did not differ significantly.

The relatively higher EC under KH and RHB + KH may be attributed to the addition of soluble ionic constituents supplied by KH, particularly K^+^ and other soluble inorganic and organic ions (Table 1), which increase the ionic strength of the soil solution. The similar EC values under KH and RHB + KH suggest that the presence of KH contributed substantially to the higher EC observed in these treatments, although the significant RHB × KH interaction indicates that the effects of the two amendments were not simply additive. Conversely, the lower EC under RHB is consistent with previous studies reporting that RHB can influence soil EC through its porous structure, high specific surface area, oxygen-containing functional groups, and interactions with dissolved ions [33]. These properties may enhance ion retention through adsorption, cation exchange, or precipitation processes, thereby reducing the concentration of freely dissolved ions in the soil solution.

**Table 1:** Initial properties of soil and amendments prior to use for the experiment.

Property	Soil	Amendment	
		RHB	KH
Soil texture	Clay loam	–	–
pH value (-)	5.5 (0.1) ^1^	6.8 (0.0)	10.8 (0.1)
Electrical conductivity (EC, mS/cm)	0.3 (0.0)	0.1(0.0)	10.8 (0.2)
Total dissolved solids (ppm)	158.3 (21.3)	–	–
Organic matter (OM, %)	6.2 (0.2)	12.6 (1.6)	36.7 (0.8)
Organic carbon (OC, %)	3.6 (0.1)	7.3 (0.9)	21.3 (0.5)
Total nitrogen (TN, mg/kg)	1266.7 (163.3)	4350.5 (90.7)	3473.6 (89.7)
Available phosphorus (P, mg/kg)	42.2 (14.0)	74.0 (6.1)	75.3 (19.2)
Available potassium (K, mg/kg)	117.2 (16.2)	2610.7 (167.5)	7276.9 (771.4)
Humic acid (%) ^2^	–	–	67.5 (2.5)
Water solubility (%) ^2^	–	–	97.5 (2.5)

^1^ Values in parentheses are standard deviations. ^2^ Values that appear on the product label.

The relatively small absolute differences in EC indicate that the amendments caused only modest changes in soil EC under the conditions of this study. Similar responses have been reported in previous studies, where biochar and humic substances caused only slight increases or decreases in soil EC depending on amendment characteristics, soil properties, and environmental conditions [29,34,35].

#### 2.1.2. Total Soil Nitrogen

Post-harvest total soil N (TN) differed among treatments (Figure 1c). Factorial ANOVA indicated significant main effects of RHB and KH (both *p* < 0.001), whereas the RHB × KH interaction was not significant (Table A1). As shown on the left *y*-axis of Figure 1c, TN increased under the KH (1550 mg/kg) and RHB + KH (1650 mg kg) treatments by 11% and 18%, respectively, compared with the control (1400 mg/kg). Because both KH-containing treatments exhibited higher TN than the control, the results indicate that KH application contributed substantially to the higher residual soil TN observed at harvest. The higher TN under RHB + KH than under KH alone was numerical, and the nonsignificant RHB × KH interaction provides no evidence of a synergistic effect of the two amendments on TN.

The increase in TN under KH may partly reflect direct N input from the amendment, including NO_3_^−^-N (Table 1). However, the amount of N supplied by KH alone is unlikely to fully account for the observed increase in soil TN. Humic substances contain carboxyl and phenolic functional groups that increase soil CEC and enhance the retention of positively charged N species, particularly NH_4_^+^, thereby reducing their mobility and potential loss [28]. Although these exchange sites do not directly retain NO_3_^−^, humic substances may indirectly promote nitrate persistence by improving soil aggregation, stimulating microbial immobilization, and facilitating the incorporation of mineral N into microbial biomass and soil OM. In addition, humate amendments have been reported to influence nitrification and denitrification processes, thereby improving N retention and reducing N losses through leaching or gaseous emissions [36,37,38]. These processes may have contributed to the greater residual soil TN observed in the KH-treated soils.

The higher TN observed under RHB + KH relative to the control suggests that the combined treatment favored residual soil N accumulation at harvest. Previous studies have reported that biochar can retain NH_4_^+^ through surface adsorption and cation exchange and can provide microsites that influence microbial N transformations [39,40]. Humic substances may contribute organic N and enhance interactions between mineral N, microbial biomass, and soil organic matter [29]. When applied together, these amendments may therefore create complementary conditions for N retention; however, the nonsignificant RHB × KH interaction (*p* = 0.097) indicates that the present study provides no statistical evidence of a synergistic effect on soil TN. Similar increases in soil TN have been reported following the combined application of biochar and humic substances in rice-based systems [27,41].

In contrast, RHB alone did not significantly affect TN relative to the control, indicating that biochar application was insufficient to increase residual soil TN when applied alone under the conditions of this study. This treatment-level comparison does not conflict with the significant RHB main effect in the factorial ANOVA because the main effect represents the average effect of RHB across both KH levels.

#### 2.1.3. Soil Mineral Nitrogen Status

The soil mineral N composition at harvest reflected treatment-associated differences together with the anaerobic conditions characteristic of flooded paddy soils (Figure 1c). Under restricted oxygen availability, nitrification is limited and NH_4_^+^ generally becomes the predominant mineral N form, whereas NO_3_^−^ is readily consumed through reductive processes such as denitrification and dissimilatory nitrate reduction to ammonium (DNRA) [42,43].

Residual soil NO_3_^−^-N differed among treatments (Figure 1c). Factorial ANOVA indicated a significant RHB main effect and RHB × KH interaction (both *p* < 0.001), whereas the KH main effect was not significant (*p* = 0.234; Table A1). As shown on the right *y*-axis of Figure 1c, the control had the highest NO_3_^−^ concentration (3.32 mg/kg), whereas significantly lower concentrations were observed under RHB (0.04 mg/kg), RHB + KH (1.53 mg/kg), and KH (1.82 mg/kg), corresponding to reductions of 99%, 54%, and 45%, respectively. Thus, amendment application altered the residual NO_3_^−^ pool at harvest, with the strongest reduction occurring under RHB.

The very low NO_3_^−^ concentration under RHB may reflect biochar-induced changes in N retention and transformation. Biochar can alter soil aeration, redox conditions, microbial habitats, and dissolved organic carbon availability, thereby influencing processes such as microbial N immobilization, denitrification, and DNRA [44,45]. These processes provide plausible explanations for the lower residual extractable NO_3_^−^ concentration observed under RHB. In addition, although NO_3_^−^ is highly soluble and generally weakly retained by negatively charged soil exchange sites [46], its persistence may be indirectly influenced by biochar-induced changes in soil structure, water movement, and microbial activity.

The intermediate NO_3_^−^ concentrations under KH and RHB + KH may reflect differences in N transformation and acquisition within the soil–plant system. Humic substances have been reported to stimulate root development, nutrient acquisition, and microbial activity, potentially influencing the distribution of N among soil, microbial, and plant pools [47,48]. The lower residual NO_3_^−^ concentration under RHB + KH than under KH alone, together with the significant RHB × KH interaction, indicates that the effect of RHB on residual NO_3_^−^ depended on the presence of KH. However, the underlying N transformation processes were not directly measured in the present study.

Residual soil NH_4_^+^-N also differed among treatments, with factorial ANOVA indicating significant RHB, KH, and RHB × KH effects (all *p* < 0.001; Table A1). The soil NH_4_^+^ concentration was significantly higher under KH (19.58 mg/kg) than in the control (16.44 mg/kg), representing a 19% increase, whereas RHB and RHB + KH maintained NH_4_^+^ concentrations comparable to the control (Figure 1c). Humic functional groups, particularly carboxyl and phenolic groups, can contribute to cation exchange and organo-mineral interactions that favor NH_4_^+^ retention [21]. Humic substances may also modify microbial N transformations under flooded conditions and thereby influence the balance between mineral N forms [49,50]. However, these processes are considered possible mechanisms rather than demonstrated explanations for the observed NH_4_^+^ patterns.

Amendment-derived nutrient inputs provide additional context for interpreting these N responses (Table A2). Based on the measured amendment composition and application rates, RHB supplied approximately 65.26 kg/ha of total N, whereas KH supplied only 0.022 kg/ha. Thus, responses under RHB may partly reflect direct N addition as well as changes in soil N availability and plant acquisition. In contrast, the negligible N input from KH indicates that its effects on soil N status and shoot N accumulation are unlikely to be explained by direct N addition alone. Nevertheless, total N content does not indicate the fraction released or available during the growing season, and the nutrient contributions of the incorporated green manure and cow manure were not quantified. Therefore, the relative contributions of background organic nutrient inputs, direct amendment-derived N, changes in soil N availability, and plant acquisition cannot be quantitatively separated.

Although RHB + KH produced the highest soil TN, extractable NH_4_^+^ remained near the control level, while NO_3_^−^ was lower at harvest. KH alone resulted in higher residual soil TN and NH_4_^+^, whereas RHB was associated with a marked reduction in NO_3_^−^. These contrasting post-harvest patterns indicate treatment-associated differences in total and extractable mineral N pools at harvest but do not demonstrate transformation or transfer among these pools. Biochar has been reported to retain NH_4_^+^ through adsorption and cation exchange and to influence its incorporation into less readily extractable microbial or organic N pools [39,40,51], which may provide possible mechanisms for the observed pattern under RHB + KH.

#### 2.1.4. Available Phosphorus

Post-harvest soil available P differed among treatments (Figure 1d). Factorial ANOVA indicated significant effects of RHB, KH, and their interaction (all *p* < 0.001; Table A1). Available P decreased under RHB (33.5 mg/kg), KH (48.7 mg/kg), and RHB + KH (47.2 mg/kg) relative to the control (106.2 mg/kg), corresponding to reductions of 68.5%, 54.1%, and 55.6%, respectively (Figure 1d).

The lower extractable P observed under RHB may reflect changes in P partitioning and extractability during the growing season. Previous studies have shown that biochar can influence soil P dynamics through adsorption, surface complexation, and precipitation reactions involving Ca-, Fe-, and Al-containing minerals, potentially redistributing P among forms with different extractabilities [52,53]. Greater shoot P accumulation under RHB (Section 2.2) may also have contributed to the lower residual extractable P pool at harvest.

Similarly, the lower post-harvest extractable P under KH may be associated with changes in P partitioning within the soil–plant system. Humic substances can interact with Ca–P and Fe–P phases and promote organo-mineral complex formation, thereby influencing P availability [54]. Under flooded conditions, interactions between humic substances, Fe redox chemistry, and Fe–P associations may further modify P behavior [21,55]. However, these processes are considered possible mechanisms rather than demonstrated explanations for the observed available P patterns.

The RHB + KH treatment showed a reduction in available P similar to that observed under KH alone. This non-additive response is consistent with the significant RHB × KH interaction (*p* < 0.001; Table A1), indicating that the effect of RHB on available P depended on the presence of KH. Consideration of the amendment-derived P inputs (Table A2) indicates that the residual soil P responses cannot be interpreted solely from amendment P addition. The lower post-harvest available P across the amended treatments may reflect differing contributions from changes in P extractability, plant acquisition, and redistribution among soil P pools. Because initial plot-level available P and seasonal P balances were not determined, their relative contributions cannot be quantitatively distinguished.

#### 2.1.5. Exchangeable Potassium

Post-harvest soil exchangeable K differed among treatments (Figure 1d). Factorial ANOVA indicated significant main effects of RHB and KH (both *p* < 0.001), whereas the RHB × KH interaction was not significant (*p* = 0.392; Table A1). RHB application (107.0 mg/kg) resulted in a 10% reduction compared with the control (118.9 mg/kg). The lower residual exchangeable K under RHB may reflect differences in K availability and plant acquisition during the growing season. Previous studies have shown that biochar can influence K dynamics through cation exchange, adsorption–desorption processes, and interactions with surface functional groups and pore structures [56]. The greater shoot K accumulation under RHB (Section 2.2) may also have contributed to the lower exchangeable K remaining in the soil at harvest.

Exchangeable K under KH and RHB + KH was comparable to that of the control. Although KH had a relatively high available K concentration (Table 1), its low application rate resulted in only a small direct K input (Table A2). Thus, the absence of higher residual exchangeable K under KH is consistent with the limited quantity of amendment-derived K added to the soil. Previous studies have reported increased exchangeable K following KH application in pot experiments [21] and under mineral fertilizer-based K management in rice systems [57], suggesting that responses may depend on amendment rate and soil and management conditions.

Co-application of RHB and KH likewise did not increase the residual exchangeable K pool relative to the control. This is consistent with the nonsignificant RHB × KH interaction (*p* = 0.392), which provides no evidence of an interactive effect of the two amendments on exchangeable K. Biochar has been reported to influence K retention and release through adsorption–desorption and cation-exchange processes [58,59], while plant acquisition can further affect the amount remaining in exchangeable form at harvest. Overall, the observed treatment differences indicate changes in the residual exchangeable K pool rather than providing evidence for a specific K-retention or transformation mechanism.

The mechanisms discussed above represent plausible interpretations based on processes reported in previous studies rather than processes directly quantified in the present experiment. Accordingly, the observed N, P, and K responses are interpreted primarily as treatment-associated differences in measured soil nutrient pools, while the relative contributions of microbial transformations, adsorption–desorption, precipitation, plant acquisition, and other nutrient processes remain to be resolved.

### 2.2. Shoot Nutrient Accumulation Response to Amendments

**Figure 2:**
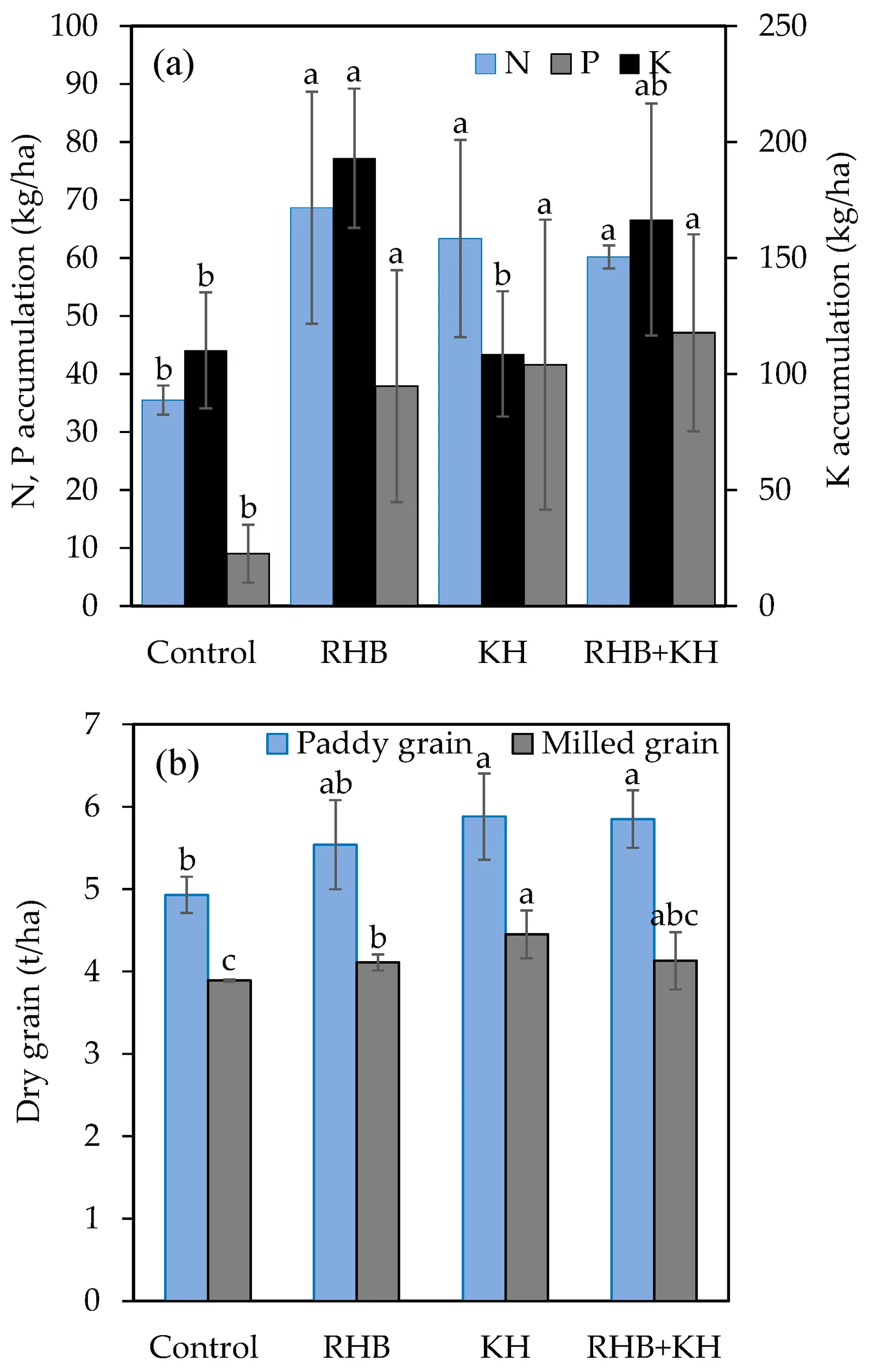
(**a**) Shoot accumulation of nitrogen (N), phosphorus (P), and potassium (K), and (**b**) paddy and milled rice yields at 14% moisture content. Values are means ± standard deviation (SD; *n* = 4). Different lowercase letters indicate significant differences among treatments (*p* < 0.05).

Amendment treatments were associated with changes in shoot nutrient accumulation under flooded conditions. Shoot N accumulation differed among treatments (Figure 2a). Factorial ANOVA indicated significant effects of RHB (*p* < 0.001), KH (*p* = 0.002), and their interaction (*p* < 0.001; Table A1). Shoot N accumulation increased markedly under RHB (68.6 kg/ha), KH (63.4 kg/ha), and RHB + KH (60.2 kg/ha) compared with the control (35.5 kg/ha), corresponding to increases of 93.3%, 78.4%, and 69.4%, respectively. The RHB + KH treatment also showed the lowest variability in shoot N accumulation among replicates.

The greater shoot N accumulation under RHB may reflect both direct amendment-derived N input and changes in soil N availability. As discussed in Section 2.1.3, RHB provided a substantial direct N input, although the fraction released and available during the growing season was not determined. Biochar has also been reported to influence N retention and availability in flooded soils through NH_4_^+^ adsorption, changes in microbial habitats, and other soil processes affecting N availability [60,61,62]. In contrast, the direct N input from KH was negligible (Table A2), indicating that the substantial increase in shoot N accumulation under KH cannot be explained by direct N addition alone. Humic substances have been reported to promote root development and physiological processes associated with nutrient acquisition and N assimilation [28,63]. Nevertheless, because seasonal N fluxes and amendment-derived N accumulation in shoots were not measured separately, the relative contributions of direct N addition and changes in N availability or acquisition cannot be quantified.

Shoot P accumulation also differed among treatments (Figure 2a). Factorial ANOVA indicated significant effects of RHB (*p* = 0.018), KH (*p* < 0.001), and their interaction (*p* = 0.021; Table A1). Shoot P accumulation increased under RHB + KH (47.1 kg/ha), KH (41.6 kg/ha), and RHB (37.9 kg/ha) compared with the control (9.0 kg/ha), corresponding to increases of 423.3%, 362.2%, and 321.1%, respectively. These increases occurred together with lower residual extractable soil P at harvest (Section 2.1.4), but the two observations do not establish that the decrease in soil extractable P resulted from shoot P accumulation. As discussed in Section 2.1.4, initial plot-level available P, soil P fractions, and seasonal P balances were not determined, and the amendment-derived P estimates in Table A2 represent available rather than total P inputs.

The greater shoot P accumulation in the amended treatments is consistent with previous studies reporting changes in plant P nutrition following biochar or humic substance application [17,64]. Biochar can modify soil chemical conditions and phosphate interactions with Fe and Al minerals, particularly in acidic soils [64], whereas humic substances can influence P availability through interactions with soil minerals and organic matter and through effects on root development [17,65]. These processes provide possible explanations for the observed shoot P responses but were not directly evaluated in the present study.

Shoot K accumulation also differed among treatments (Figure 2a). Factorial ANOVA indicated a significant RHB effect (*p* < 0.001), whereas neither the KH main effect (*p* = 0.483) nor the RHB × KH interaction (*p* = 0.616) was significant (Table A1). RHB substantially increased shoot K accumulation (193.0 kg/ha), representing a 75.1% increase relative to the control (110.2 kg/ha), whereas shoot K accumulation under RHB + KH (166.5 kg/ha) and KH (108.7 kg/ha) did not differ significantly from the control.

The greater shoot K accumulation under RHB may reflect both amendment-derived K and biochar-induced changes in soil K availability, as discussed in Section 2.1.5. Biochar has been reported to influence K availability through cation exchange and other soil chemical processes [65,66,67]. In contrast, despite the relatively high available K concentration of KH (Table 1), its low application rate resulted in only a small direct available K input (Table A2), which may partly explain the absence of a significant increase in shoot K accumulation under KH.

Overall, the amendments produced nutrient-specific changes in shoot N, P, and K accumulation. When considered together with the amendment-derived nutrient inputs (Table A2), the responses suggest that direct nutrient addition may have contributed to the effects of RHB, whereas the comparatively small nutrient inputs from KH indicate that its effects, particularly on shoot N accumulation, cannot be explained by direct nutrient supply alone.

### 2.3. Yield Performance

Paddy and milled grain yields differed among treatment combinations (Figure 2b). Factorial ANOVA indicated a significant main effect of KH on both paddy (*p* = 0.013) and milled grain yield (*p* = 0.023), whereas the RHB main effect was not significant for either paddy (*p* = 0.188) or milled grain yield (*p* = 0.207). The RHB × KH interaction was also not significant for paddy (*p* = 0.077) or milled grain yield (*p* = 0.164; Table A1). The KH and RHB + KH treatments produced the highest paddy grain yields (5.88 and 5.85 t/ha, respectively), representing increases of 19.3% and 18.7% relative to the control (4.93 t/ha), whereas RHB (5.54 t/ha) did not significantly differ from the control. For milled rice, KH produced the highest yield (4.45 t/ha), corresponding to a 14.4% increase relative to the control (3.89 t/ha), while RHB increased milled rice yield to 4.11 t/ha, representing a 5.7% increase. Although RHB + KH produced a high paddy grain yield, its milled rice yield (4.13 t/ha) did not differ significantly from the control. The contrasting responses of paddy and milled yield indicate that increases in harvested grain mass were not necessarily accompanied by proportional increases in the amount of rice recovered after milling. Thus, KH was the only treatment that significantly increased both paddy and milled rice yields relative to the control, whereas RHB increased milled yield without a significant increase in paddy yield.

The greater yields under KH were accompanied by higher soil TN and NH_4_^+^ concentrations (Section 2.1), greater plant N and P accumulation (Section 2.2), and higher grain N and protein contents (Section 2.4.2). Humic substances contain carboxyl and phenolic functional groups that can influence nutrient retention and availability and have been reported to promote plant nutrient acquisition [17]. The concurrence of these responses with higher paddy and milled rice yields under KH is consistent with previous studies reporting improved rice productivity following humic amendment application. Similar yield increases have been reported following Si–humate application in acid sulfate soils, where improved soil chemical conditions and P nutrition were associated with greater rice productivity [24].

RHB increased milled rice yield despite not significantly increasing paddy yield relative to the control. This pattern may reflect differences in milling recovery rather than an increase in total harvested grain mass. Greater plant N, P, and K accumulation under RHB (Section 2.2) and its effects on soil pH (Section 2.1) occurred alongside the increase in milled yield. Previous studies have similarly reported positive yield responses to biochar associated with changes in soil chemical conditions and nutrient retention [68,69]. In the present study, however, the RHB response was more evident for milled rice yield than for paddy yield. Nevertheless, factorial ANOVA showed no significant overall RHB main effect on either yield measure (Table A1).

The combined RHB + KH treatment produced a high paddy yield but did not significantly increase milled rice yield relative to the control. Thus, the greater paddy yield under the combined treatment was not accompanied by a corresponding increase in milled rice yield. Although both amendments influenced soil nutrient status and plant nutrient accumulation, the RHB × KH interaction was not significant for either paddy or milled grain yield. Therefore, the yield response of the combined treatment does not provide evidence of a synergistic effect between RHB and KH. Responses to combined biochar and humic amendments have also been reported in other crops [27,70], although their effects may vary with crop, amendment rate, soil conditions, and management practices.

Taken together, the yield responses differed between harvested paddy and the final milled product. KH increased both yield measures, RHB increased milled yield without significantly affecting paddy yield, and RHB + KH increased paddy yield without a corresponding significant increase in milled yield. These contrasting responses emphasize the value of evaluating both paddy and milled rice yields when assessing amendment effects on rice productivity and marketable grain output.

### 2.4. Rice Grain Properties

#### 2.4.1. Grain Color and Cooking Quality

The application of RHB and KH influenced several rice grain appearance and cooking quality attributes (Table 2). Among the color parameters, grain L* differed significantly among treatment combinations, with the highest value observed under RHB + KH, indicating a brighter grain appearance than the control. Factorial ANOVA indicated significant effects of RHB and KH and a significant RHB × KH interaction (Table A1). Grain lightness is an important visual quality attribute that contributes to consumer perception and market preference, with higher L* values generally reflecting a brighter grain surface [71,72]. Although a* values also differed significantly among treatments, the magnitude of the differences was small, indicating only minor changes in grain redness. Factorial ANOVA indicated a significant KH effect and RHB × KH interaction for a*, whereas the RHB main effect was not significant (Table A1). In contrast, KH produced the lowest b* value, indicating reduced grain yellowness, whereas RHB resulted in the highest b* value, suggesting a slightly more yellow grain appearance (Table 2). Significant RHB and KH effects and a significant RHB × KH interaction were detected for b* (Table A1). These results indicate that the amendments modified grain color, with RHB + KH producing the brightest grains and KH reducing grain yellowness.

Amendment application resulted in numerical variation in several cooking quality attributes (Table 2). The volumetric expansion ratio was numerically higher in the amended treatments than in the control, with the highest mean observed under RHB, followed by RHB + KH, KH, and the control. Factorial ANOVA detected a significant RHB main effect on volumetric expansion ratio (*p* = 0.046), whereas the KH main effect and RHB × KH interaction were not significant (Table A1). Similarly, KH exhibited the highest numerical grain elongation ratio, but no significant factorial effects were detected. Cooking time and water uptake ratio also remained unaffected by RHB, KH, or their interaction. Similar variation in rice cooking characteristics has been reported under different nutrient-management conditions, potentially reflecting differences in grain composition [73]. Thus, the volumetric expansion response was associated with a significant RHB effect, whereas the observed variation in grain elongation, cooking time, and water uptake was not supported by significant factorial effects.

Gel consistency remained below 40 mm in all treatments and did not differ significantly among treatment combinations, indicating that the rice maintained a hard gel consistency regardless of amendment application. Factorial ANOVA likewise detected no significant RHB, KH, or RHB × KH effects on gel consistency (Table A1). The absence of significant effects indicates that the amendments did not measurably alter this textural characteristic of the rice under the conditions of the study. Gel consistency is widely used to evaluate the texture of cooked rice after cooling, with lower values corresponding to firmer gels and higher values indicating softer gels [72].

#### 2.4.2. Proximate and Nutritional Compositions

Soil amendments influenced several components of the proximate and nutritional composition of rice grains, although the magnitude and statistical pattern of the effects differed among components (Table 2; Table A1). Carbohydrate content was highest in the control (89.2%) and decreased under RHB (88.7%), KH (87.9%), and RHB + KH (88.3%). Factorial ANOVA showed significant effects of RHB and KH and a significant RHB × KH interaction on carbohydrate content (all *p* < 0.001; Table A1). These reductions coincided with increases in grain protein and fat contents, indicating a shift in grain macronutrient composition rather than a uniform improvement in grain quality. Similar trade-offs have been reported under improved N nutrition, where enhanced amino acid and protein synthesis can compete with starch accumulation for available carbon skeletons [74,75]. Although the absolute differences in carbohydrate content were relatively small (0.5–1.3 percentage points relative to the control), the significant decreases were included in the overall assessment of treatment effects on grain composition rather than being interpreted as secondary to the increases in protein and fat.

Consistent with this pattern, protein content increased from 9.9% in the control to 10.2%, 11.0%, and 10.6% under RHB, KH, and RHB + KH, respectively. For protein content, factorial ANOVA showed a significant KH effect (*p* < 0.001) and RHB × KH interaction (*p* < 0.001), whereas the RHB main effect was not significant (*p* = 0.377; Table A1). These changes corresponded to higher grain N concentrations, which increased from 1.66% in the control to 1.71%, 1.84%, and 1.78% under RHB, KH, and RHB + KH, respectively. A similar statistical pattern was observed for grain N, with a significant KH effect and RHB × KH interaction (both *p* < 0.001), but no significant RHB main effect (*p* = 0.379; Table A1). The greater shoot N accumulation observed in the amended treatments and higher residual soil TN under KH and RHB + KH were accompanied by higher grain N concentrations, although these concurrent responses do not establish a direct nutrient-transfer pathway. Similar increases in grain protein following biochar and humic substance application have been reported previously and have been associated with enhanced N acquisition and assimilation during grain filling [29,76,77]. From a nutritional perspective, the increase in grain protein was modest, with the largest absolute increase observed under KH (+1.1 percentage points), indicating a measurable change in grain nutritional composition. However, higher protein content does not necessarily represent superior overall grain quality, particularly for premium aromatic rice, for which appearance, cooking, textural, and sensory characteristics are also important. Therefore, the observed increase in protein should be interpreted as a nutritional compositional response rather than an overall improvement in consumer-preferred grain quality.

Grain fat content also varied among treatment combinations, rising from 0.38% in the control to 0.55%, 0.53%, and 0.48% under RHB, KH, and RHB + KH, respectively. Factorial ANOVA indicated a significant RHB effect (*p* = 0.018) and RHB × KH interaction (*p* < 0.001), whereas the KH main effect was not significant (*p* = 0.076; Table A1). These changes, together with the concurrent decrease in carbohydrate content, further demonstrate that amendment effects involved differences among grain macronutrient fractions rather than uniformly favorable changes in all compositional attributes. Potassium has important roles in carbohydrate transport, enzyme activation, and plant metabolic processes during grain development [78], which may provide a physiological context for the observed compositional differences.

Ash content also varied among treatment combinations, with the highest value under RHB (0.70%), followed by RHB + KH (0.60%), KH (0.56%), and the control (0.51%). Factorial ANOVA showed a significant RHB effect (*p* = 0.001) and RHB × KH interaction (*p* = 0.008), whereas the KH main effect was not significant (*p* = 0.286; Table A1). This response is consistent with the higher concentrations of some mineral nutrients measured in the grains and with the greater shoot nutrient accumulation observed for several treatment–nutrient combinations (Section 2.2). Biochar provides a direct source of mineral nutrients, whereas humic substances have been widely reported to improve nutrient availability and uptake [79]. The higher ash values in the amended treatments may therefore be consistent with changes in grain mineral composition; however, ash content represents total inorganic residue and does not by itself identify the minerals responsible for the increase.

Moisture content also varied among treatments, decreasing to 13.22% under RHB and increasing to 14.13% under RHB + KH compared with 13.60% in the control. Factorial ANOVA showed a significant KH effect and RHB × KH interaction (both *p* < 0.001), whereas the RHB main effect was not significant (*p* = 0.390; Table A1). Although these differences were statistically significant among treatment combinations, the magnitude of variation was relatively small, indicating that amendment application had only a limited influence on grain moisture content.

Grain P and K concentrations showed clear treatment-associated differences that occurred alongside the changes in shoot nutrient accumulation. The P concentration increased from 0.10% in the control to 0.14%, 0.12%, and 0.13% under RHB, KH, and RHB + KH, respectively, while the K concentration increased from 0.06% to 0.11%, 0.09%, and 0.13%. These increases occurred alongside the greater P and K accumulation measured in rice shoots (Section 2.2) despite the lower residual extractable P and exchangeable K remaining in the soil at harvest (Section 2.1). Factorial ANOVA indicated significant main effects of both RHB and KH on grain P and K concentrations, whereas their interactions were not significant (Table A1). These concurrent responses indicate treatment-associated differences across soil, shoot, and grain nutrient measurements; however, they do not establish the direction or magnitude of nutrient transfer among these compartments. Phosphorus plays essential roles in energy metabolism and biosynthetic reactions, whereas K supports assimilate transport and enzyme-mediated processes involved in grain filling [80].

Zn and amylose contents also differed among treatments. The Zn concentration ranged from 2.13 to 2.48 mg/100 g DM. RHB did not differ significantly from the control, whereas KH and RHB + KH had significantly lower Zn concentrations, with KH showing the lowest value. Factorial ANOVA showed significant main effects of RHB (*p* = 0.003) and KH (*p* < 0.001), but no significant RHB × KH interaction (*p* = 0.310; Table A1). Amylose content decreased from 18.5% in the control to 17.7%, 17.6%, and 17.2% under RHB, KH, and RHB + KH, respectively. All amended treatments had significantly lower amylose contents than the control but did not differ significantly from one another. Factorial ANOVA likewise showed significant main effects of RHB (*p* = 0.002) and KH (*p* < 0.001), with no significant interaction (*p* = 0.202; Table A1). Although amylose content is strongly influenced by genotype [75], the present results indicate that amendment treatments were associated with modest but significant differences in grain amylose content. Zn accumulation is regulated by physiological transport and homeostatic mechanisms within the plant [81], although these processes were not directly evaluated in the present study.

The changes in grain composition observed in the amended treatments were accompanied by differences in soil nutrient status and shoot nutrient accumulation. Grain protein, fat, ash, P, and K contents generally increased under amendment application, whereas carbohydrate and amylose contents decreased. Grain Zn showed a treatment-specific response, remaining statistically comparable to the control under RHB but decreasing under KH and RHB + KH. Thus, the compositional response was parameter-specific rather than representing a uniform improvement in grain nutritional quality. The higher grain N and protein contents occurred alongside greater shoot N accumulation (Section 2.2) and differences in soil N status (Section 2.1), while changes in grain P and K were similarly accompanied by treatment-associated differences in soil and shoot nutrient measurements. These concurrent responses indicate associations among soil, shoot, and grain nutrient status but do not establish direct nutrient-transfer pathways among these compartments.

Overall, RHB and KH altered the proximate and nutritional composition of KDML105 grain, with increases in selected nutritional components occurring alongside decreases in other attributes. The increases in protein, fat, ash, P, and K indicate significant compositional responses to amendment application, while carbohydrate and amylose contents decreased and Zn exhibited a treatment-specific response. Accordingly, the effects of RHB and KH on grain composition should be interpreted as treatment- and parameter-specific changes rather than as a generalized improvement in nutritional quality.

#### 2.4.3. Bioactive Compounds

In parallel with the changes in proximate and mineral composition, soil amendments significantly influenced the bioactive composition of rice grains, as reflected by differences in DPPH radical-scavenging activity and total phenolic content (TPC) among treatment combinations (Table 2). These responses indicate treatment-associated differences in grain antioxidant properties and phenolic content. Factorial ANOVA further showed significant RHB and KH main effects and a significant RHB × KH interaction for DPPH activity, whereas TPC showed a significant KH effect and RHB × KH interaction but no significant RHB main effect (Table A1).

DPPH radical-scavenging activity ranged from 0.30 to 0.62 µmol Trolox eq/g DM, with the highest value observed under RHB, followed by RHB + KH and the control, whereas KH exhibited the lowest antioxidant activity. Pairwise comparisons indicated that RHB, RHB + KH, and the control did not differ significantly from one another, whereas KH had significantly lower DPPH activity than the other treatments. TPC also differed significantly among treatments, ranging from 7.62 to 9.62 mg GAE/100 g DM. The highest TPC was recorded in the control, followed by RHB + KH and RHB, whereas KH produced the lowest value, with all four treatment combinations differing significantly from one another. The differing treatment patterns for DPPH activity and TPC indicate that antioxidant capacity did not vary directly with total phenolic concentration.

Although RHB had the numerically highest DPPH activity, it did not differ significantly from the control or RHB + KH in the pairwise comparison. Nevertheless, its lower TPC relative to the control, together with comparable DPPH activity, suggests that variation in antioxidant capacity was not determined by total phenolic concentration alone. Differences in the composition and radical-scavenging potency of individual phenolic compounds, as well as contributions from other antioxidant constituents, may have influenced the response. In KDML105 rice, antioxidant capacity has been associated with several bioactive constituents, including phenolic acids, γ-oryzanol, and tocopherols, indicating that antioxidant activity reflects contributions from multiple compounds [82,83]. Biochar has also been reported to influence plant biochemical properties, including phenolic and antioxidant responses, although the magnitude and direction of these effects depend on biochar properties, soil conditions, crop species, and environmental conditions [84,85].

In contrast, KH resulted in the lowest DPPH activity and TPC despite higher values for several nutrient-related variables (Section 2.1, Section 2.2 and Section 2.4.2). Humic substances are known to influence nutrient acquisition and plant primary and secondary metabolism, with their responses depending on their chemical characteristics, concentration, and growing conditions [86,87]. The contrasting nutrient and antioxidant responses under KH may therefore reflect differences in plant metabolic responses associated with altered nutritional status rather than a direct relationship between nutrient accumulation and antioxidant production. However, because the relevant metabolic pathways and individual antioxidant compounds were not measured, this interpretation remains a possible explanation rather than a demonstrated mechanism.

The combined RHB + KH treatment produced DPPH activity intermediate numerically between RHB and KH, although it did not differ significantly from RHB or the control in the pairwise comparison. Its TPC (8.94 mg GAE/100 g DM) was significantly higher than that under either RHB or KH alone but remained significantly lower than the control. Importantly, factorial ANOVA detected significant RHB × KH interactions for both DPPH activity (*p* = 0.003) and TPC (*p* < 0.001), indicating that the effect of one amendment depended on the presence or absence of the other. Thus, the combined responses were non-additive rather than simply reflecting independent RHB and KH effects. However, the significant statistical interactions alone do not establish the biochemical mechanisms underlying these responses.

Overall, the treatment-specific responses of DPPH activity and TPC indicate that total phenolic concentration alone did not account for differences in antioxidant capacity. This is particularly evident because the control had the highest TPC but did not have the highest numerical DPPH activity, whereas RHB had lower TPC but the highest numerical DPPH activity. Differences in individual phenolic compounds and other antioxidant constituents may also contribute to the observed DPPH activity [82]. However, the present data cannot distinguish whether these differences resulted from treatment-induced changes in plant metabolism or from differences in the relative composition and concentration of grain constituents. These possibilities provide plausible explanations for the observed treatment patterns but were not directly resolved by the present measurements.

#### 2.4.4. Amino Acid Profiles

The amino acid composition of rice grain protein showed treatment-specific responses to RHB and KH (Table 2), with factorial ANOVA indicating significant RHB and/or KH main effects for some amino acids and significant RHB × KH interactions for others (Table A1). These responses indicate that the treatment-associated differences in soil nutrient status (Section 2.1), shoot nutrient accumulation (Section 2.2), and grain protein content (Section 2.4.2) were accompanied by corresponding changes in protein composition.

Non-essential amino acids, including alanine, aspartic acid, glutamic acid, glycine, and proline, showed distinct responses among treatment combinations. Glutamic acid, the most abundant amino acid reported after acid hydrolysis, increased from 3.56 g/100 g protein in the control to 3.66, 4.75, and 5.04 g/100 g protein under RHB, KH, and RHB + KH, respectively. RHB alone did not differ significantly from the control, whereas KH and RHB + KH produced higher concentrations (Table 2). Factorial ANOVA indicated a significant KH main effect and RHB × KH interaction for glutamic acid, whereas the RHB main effect was also significant but weaker (Table A1). Likewise, aspartic acid increased from 1.68 to 2.08–2.33 g/100 g protein, while glycine increased progressively from 1.58 g/100 g protein in the control to 1.88, 1.98, and 2.08 g/100 g protein under RHB, KH, and RHB + KH, respectively. Factorial ANOVA indicated significant RHB and KH main effects and a significant RHB × KH interaction for aspartic acid (Table A1). Alanine was highest under RHB + KH, while proline reached its highest concentration under RHB. Because acid hydrolysis converts glutamine and asparagine to glutamic acid and aspartic acid, respectively, the reported values should be interpreted as the corresponding acid-hydrolysis products rather than the concentrations of the native amino acids alone. These responses occurred alongside differences in soil TN (Section 2.1), shoot N accumulation (Section 2.2), and grain protein content (Section 2.4.2), although these concurrent responses do not establish a direct causal relationship. Similar responses have been reported following biochar and humic substance application under improved N nutrition [88,89].

Essential amino acids also showed strongly treatment-dependent patterns (Table 2). Leucine increased from 0.79 g/100 g protein in the control to 1.68 and 1.78 g/100 g protein under RHB and KH, respectively, but was intermediate under RHB + KH (1.09 g/100 g protein). Factorial ANOVA showed no significant RHB or KH main effect for leucine but a strong RHB × KH interaction (*p* < 0.001), indicating that the effect of one amendment depended on the presence of the other. Isoleucine decreased under RHB and KH (0.20 and 0.30 g/100 g protein, respectively) relative to the control (1.38 g/100 g protein), but increased to 1.88 g/100 g protein under RHB + KH. Consistent with this contrasting pattern, factorial ANOVA indicated a significant RHB main effect (*p* = 0.040) and a significant RHB × KH interaction (*p* < 0.001), whereas the KH main effect was not significant (Table A1). Phenylalanine was highest under RHB + KH (1.68 g/100 g protein), with significant RHB and KH main effects and a significant RHB × KH interaction. Threonine similarly decreased under RHB and KH but reached its highest concentration under RHB + KH (1.88 g/100 g protein), with a strong RHB × KH interaction (*p* < 0.001).

Lysine, typically the limiting amino acid in cereal grains, increased markedly under RHB and KH (0.89–0.99 g/100 g protein) compared with the control (0.10 g/100 g protein), whereas RHB + KH remained at the control level (0.10 g/100 g protein). Neither the RHB nor KH main effect was significant, but their interaction was highly significant (*p* < 0.001), demonstrating a non-additive response to co-application. Histidine increased from 0.10 g/100 g protein in the control to 0.40–0.50 g/100 g protein in the amended treatments, with significant RHB and KH main effects and a significant RHB × KH interaction. Valine was higher in all three amended treatment combinations (0.30 g/100 g protein) than in the control (0.10 g/100 g protein) according to the pairwise comparisons in Table 2. Factorial ANOVA also indicated significant RHB (*p* = 0.043) and KH (*p* = 0.040) main effects and a significant RHB × KH interaction (*p* = 0.042; Table A1). Tryptophan was not quantified because it is degraded during acid hydrolysis, as described in Section 3.6.4.

Sulfur-containing amino acids exhibited treatment-specific responses. Methionine increased under RHB + KH (1.09 g/100 g protein) compared with the control (0.69 g/100 g protein), but decreased under RHB (0.10 g/100 g protein) and KH (0.20 g/100 g protein). Factorial ANOVA indicated a significant KH main effect (*p* = 0.036) and a strong RHB × KH interaction (*p* < 0.001), whereas the RHB main effect was not significant. L-cystine remained unchanged at 0.198 g/100 g protein across treatments, consistent with the absence of significant RHB, KH, or RHB × KH effects (all *p* > 0.05). The greater methionine concentration under the combined treatment therefore indicates that its response depended on the amendment combination rather than representing a general increase following amendment application. Similar treatment-dependent responses in methionine have been reported under improved nutrient management, although responses vary among crop species and production systems [90,91].

Overall, the amendments produced amino-acid-specific responses rather than a uniform increase. RHB and KH increased some amino acids but decreased others, while RHB + KH produced the highest concentrations of several amino acids, with significant RHB × KH interactions indicating treatment-dependent responses. Together, these results indicate that changes in grain protein content were accompanied by changes in amino acid composition rather than a generalized increase in all measured amino acids. The frequent significant interactions further indicate that the response to one amendment often depended on whether the other amendment was present [92].

The amino acid profile further complements the treatment-associated changes observed in grain nutritional composition. Higher concentrations of selected essential amino acids were observed under particular treatment combinations—for example, lysine under RHB and KH and methionine, threonine, phenylalanine, and isoleucine under RHB + KH. However, because some essential amino acids decreased under particular treatments, the amino acid responses should not be interpreted as a uniform improvement in protein nutritional quality. Furthermore, because the present analysis quantified total amino acids following protein hydrolysis rather than free amino acids, the results should not be interpreted directly in terms of sensory attributes such as sweetness or umami perception.

The coordinated responses observed across soil nutrient dynamics (Section 2.1), shoot nutrient accumulation (Section 2.2), grain protein content (Section 2.4.2), and amino acid composition indicate that amendment application affected multiple components of the soil–plant–grain system. However, these concurrent responses represent associations among the measured compartments and do not establish direct nutrient-transfer or metabolic pathways. These findings further support the integrated soil–plant–grain framework evaluated in this study and show that the effects of RHB and KH extended beyond soil nutrient status and shoot nutrient accumulation to treatment-specific changes in the amino acid composition of the harvested grain.

### 2.5. Integrated PCA of Soil–Plant–Grain Associations

**Figure 3:**
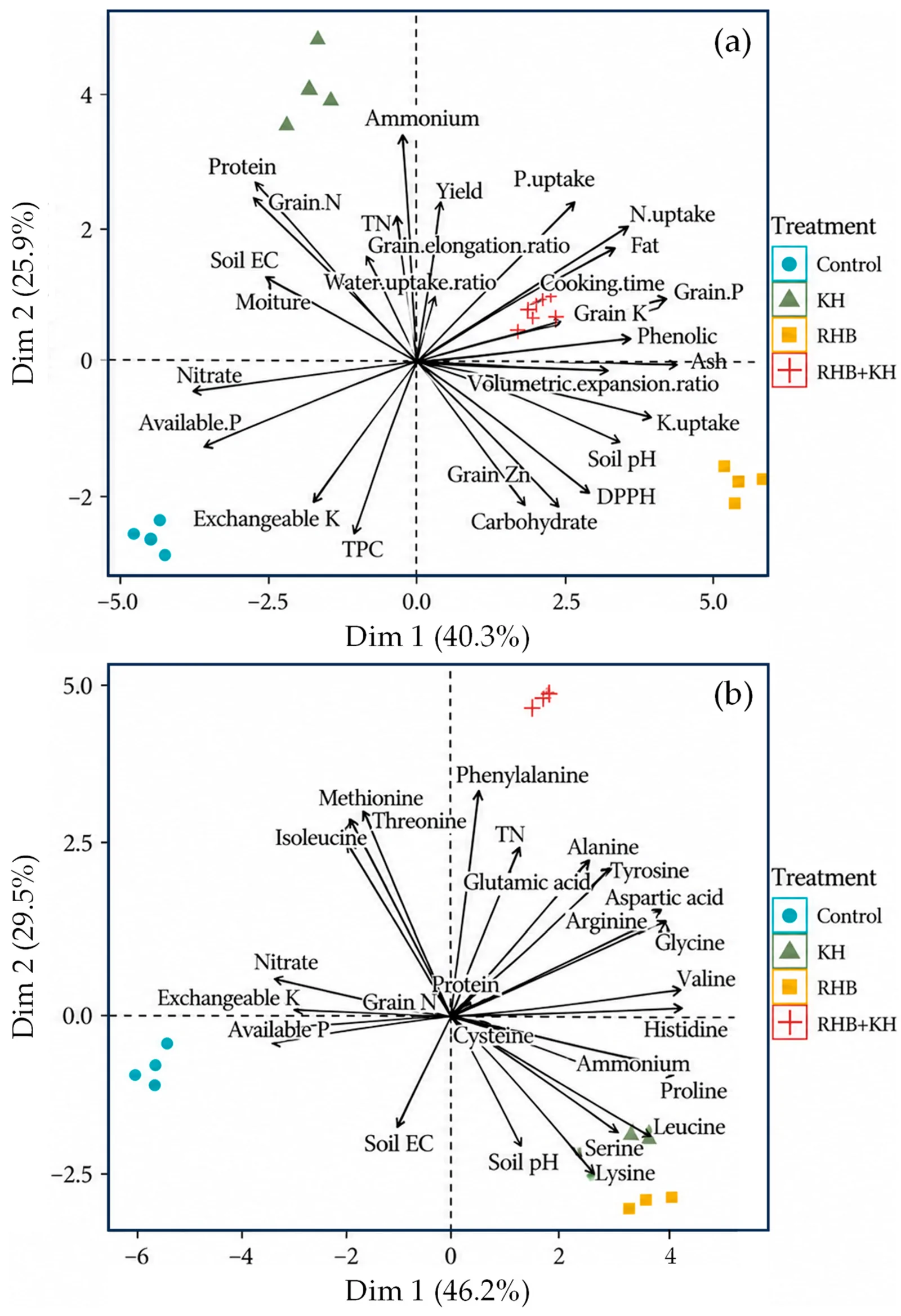
Principal Component Analysis (PCA) biplots illustrating multivariate relationships among (**a**) soil physicochemical properties, shoot nutrient accumulation, grain yield and quality traits, and (**b**) amino acid composition and nutrient status under different treatments. Symbols represent individual plot-level observations (*n* = 4 per treatment), illustrating within-treatment variation; arrows represent variable loadings on the first two principal components.

PCA revealed separation among the four treatments, with the first two principal components explaining 66.2% and 75.7% of the total variation in the soil–plant–grain quality dataset (Figure 3a) and the soil–amino acid dataset (Figure 3b), respectively. The treatment clustering reflects differences in multivariate profiles across soil, plant, and grain variables. As an exploratory analysis, PCA identifies covariance among variables but does not establish causal nutrient-transfer pathways.

In the soil–plant–grain quality dataset (Figure 3a), RHB was associated with higher soil pH, shoot K accumulation, DPPH antioxidant activity, carbohydrate content, volumetric expansion ratio, and grain Zn concentration. KH was associated with soil TN, NH_4_^+^ concentration, shoot N accumulation, grain N and protein contents, grain yield, and grain elongation ratio. These PCA associations describe the multivariate covariance structure and were broadly consistent with several of the treatment-level responses observed separately for soil properties (Section 2.1), shoot nutrient accumulation (Section 2.2), yield performance (Section 2.3), and grain composition and quality (Section 2.4).

The RHB + KH treatment occupied a distinct region of the ordination and was associated with a combination of shoot and grain nutrient variables, including shoot P accumulation and grain P and K concentrations, together with cooking-quality, bioactive, and amino-acid variables. This positioning characterizes the multivariate response profile of the combined treatment; however, significant RHB × KH interactions were identified from the factorial ANOVA and should not be inferred from proximity or separation in the PCA.

Similarly, the soil–amino acid PCA (Figure 3b) showed associations between nutrient status and grain amino acid composition, consistent with the amino-acid-specific and treatment-dependent patterns described in Section 2.4. Several amino acids showed significant RHB × KH interactions in the factorial ANOVA, but these statistical interactions are distinct from the multivariate associations visualized by PCA. Overall, the PCA integrates the patterns reported across Section 2.1, Section 2.2, Section 2.3 and Section 2.4 by visualizing the covariance structure among soil properties, shoot nutrient accumulation, and grain composition and quality, while the statistical significance of individual treatment effects and RHB × KH interactions was established by the corresponding univariate and factorial analyses.

## 3. Materials and Methods

### 3.1. Site Description

**Figure 4:**
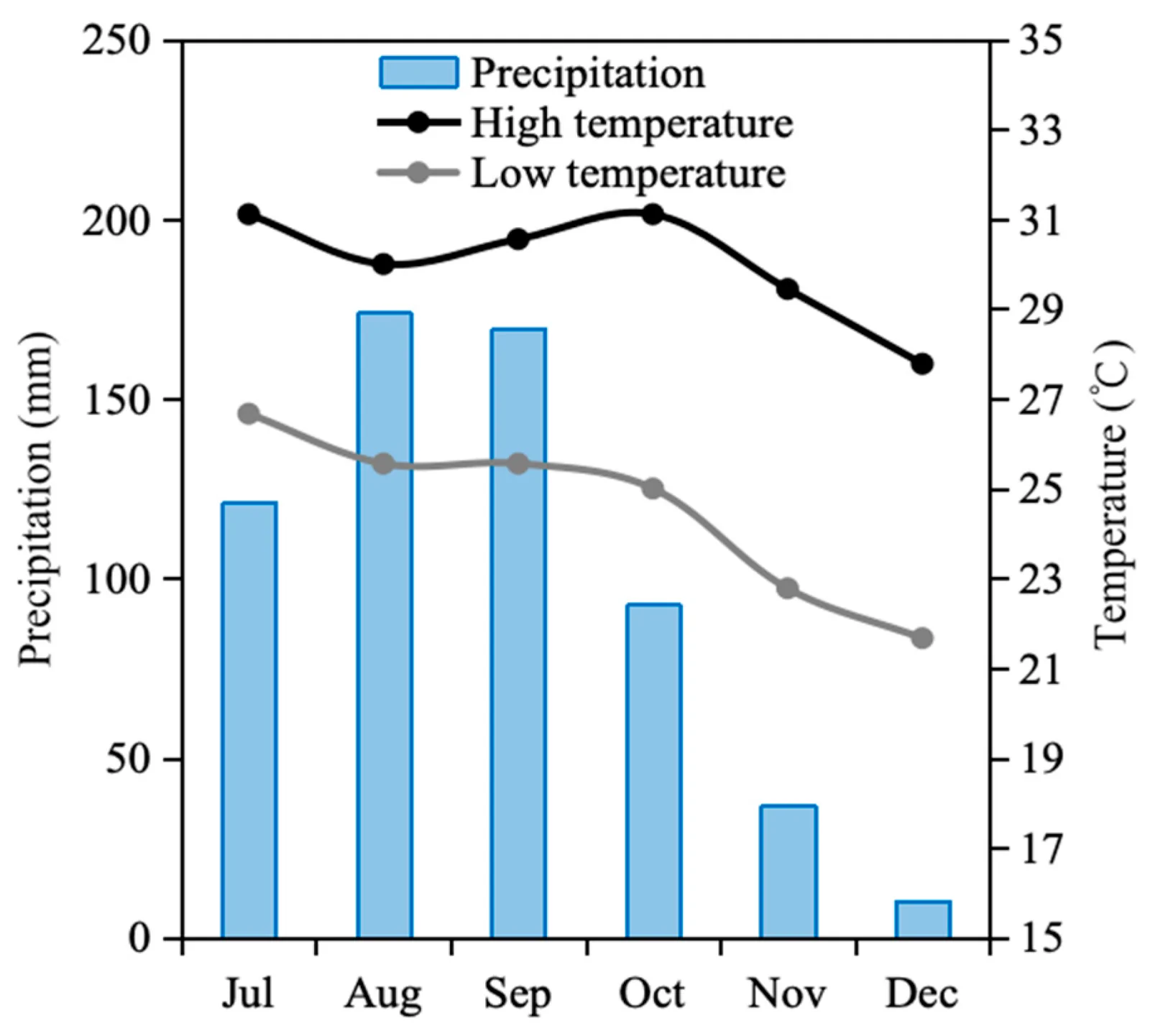
Precipitation and atmospheric temperature during the experiment.

A field experiment was conducted from July to early December 2024 in a rice paddy located in San Pa Tong, Chiang Mai, Thailand (18°38′20.7″ N, 98°50′07.4″ E). The experimental soil was classified as the San Pa Tong series (Sp; Soil Group 40), corresponding to Typic (Kandic) Paleustults according to USDA Soil Taxonomy. The area experiences a tropical savanna climate (Köppen classification: Aw) [93]. During the study period, mean daily temperatures ranged from 21.7 to 31.1 °C, with peaks exceeding 31 °C in July and October and the lowest temperatures recorded in December (Figure 4). Monthly rainfall reached its maximum in August (174.0 mm) and September (169.6 mm), and then decreased to 92.7 mm in October and 10.2 mm in December. Before treatment establishment, soil samples were collected from 10 locations across the experimental field to characterize the initial soil condition. The initial physicochemical properties of the soil and the amendments used in this study are presented in Table 1.

### 3.2. Experimental Design and Treatments

The experiment employed a randomized complete block design (RCBD) with a 2 × 2 factorial treatment structure comprising four treatments and four replicates, resulting in 16 experimental plots. The field had been continuously cultivated with rice for more than 10 years under organic management. The field was divided into four spatial blocks according to field position, with each block containing one plot of each treatment. Treatments were randomized independently within each block. This blocking arrangement was used to account for potential spatial heterogeneity across the experimental field; specific pre-existing gradients in soil fertility, drainage, or topography were not quantitatively characterized before treatment establishment. Initial soil properties were characterized at the field level but were not measured separately for each plot or block.

**Figure 5:**
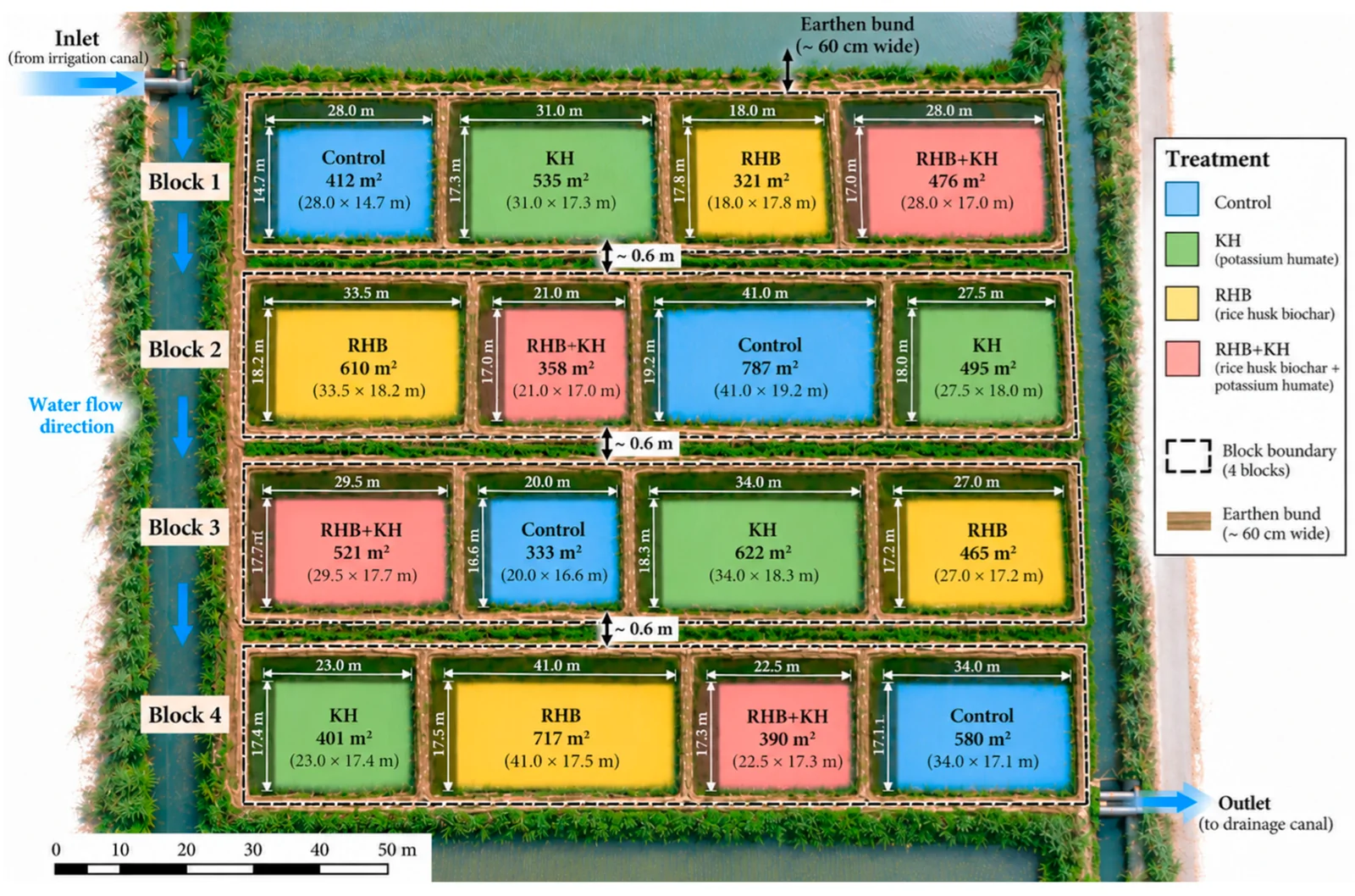
Schematic layout of the field experiment showing the four experimental blocks, spatial arrangement of the 16 plots, randomized treatment allocation within each block, plot areas, and water-flow direction. Treatments comprised the control, rice husk biochar (RHB), potassium humate (KH), and their combined application (RHB + KH). Earthen bunds approximately 60 cm wide separated adjacent plots. The layout is schematic and not drawn to scale; plot shapes and dimensions are represented approximately because the plots followed existing field boundaries and opposite sides were not necessarily equal in length.

Plot areas ranged from 321 to 787 m^2^ because the experiment was established within an existing paddy field with boundaries constrained by the existing field configuration and earthen bunds. Amendment application rates were calculated using the actual area of each plot to ensure equivalent treatment rates across plots. Despite differences in plot area, each plot constituted one experimental unit, and area-dependent response variables were standardized to a common land-area basis before analysis. Plot area was not used as a weighting factor in the statistical analysis. Block was included in the statistical model to account for spatial variation among blocks. The plots were separated by approximately 60-cm-wide earthen bunds and managed as hydraulically independent units, with no intentional water exchange between plots, to minimize movement of soluble KH and suspended RHB among treatments. The spatial arrangement of blocks and plots, treatment allocation, plot areas, and water-flow direction is shown in Figure 5.

The rice variety used was *Oryza sativa* L. cv. Khao Dawk Mali 105 (KDML105). Seeds were obtained from a local farmer’s seed stock harvested during the preceding growing season and were used uniformly across all experimental plots. The four treatments were: (1) control, no RHB or KH application; (2) RHB, 15 t/ha; (3) KH, 6.3 kg/ha; and (4) RHB + KH, the combined application of 15 t/ha RHB and 6.3 kg/ha KH. The RHB rate of 15 t/ha was selected based on application rates previously evaluated under paddy field conditions [94]. The KH rate was based on the recommended field application rate for the commercial product (1 kg/rai, equivalent to 6.25 kg/ha and rounded to 6.3 kg/ha).

**Figure 6:**
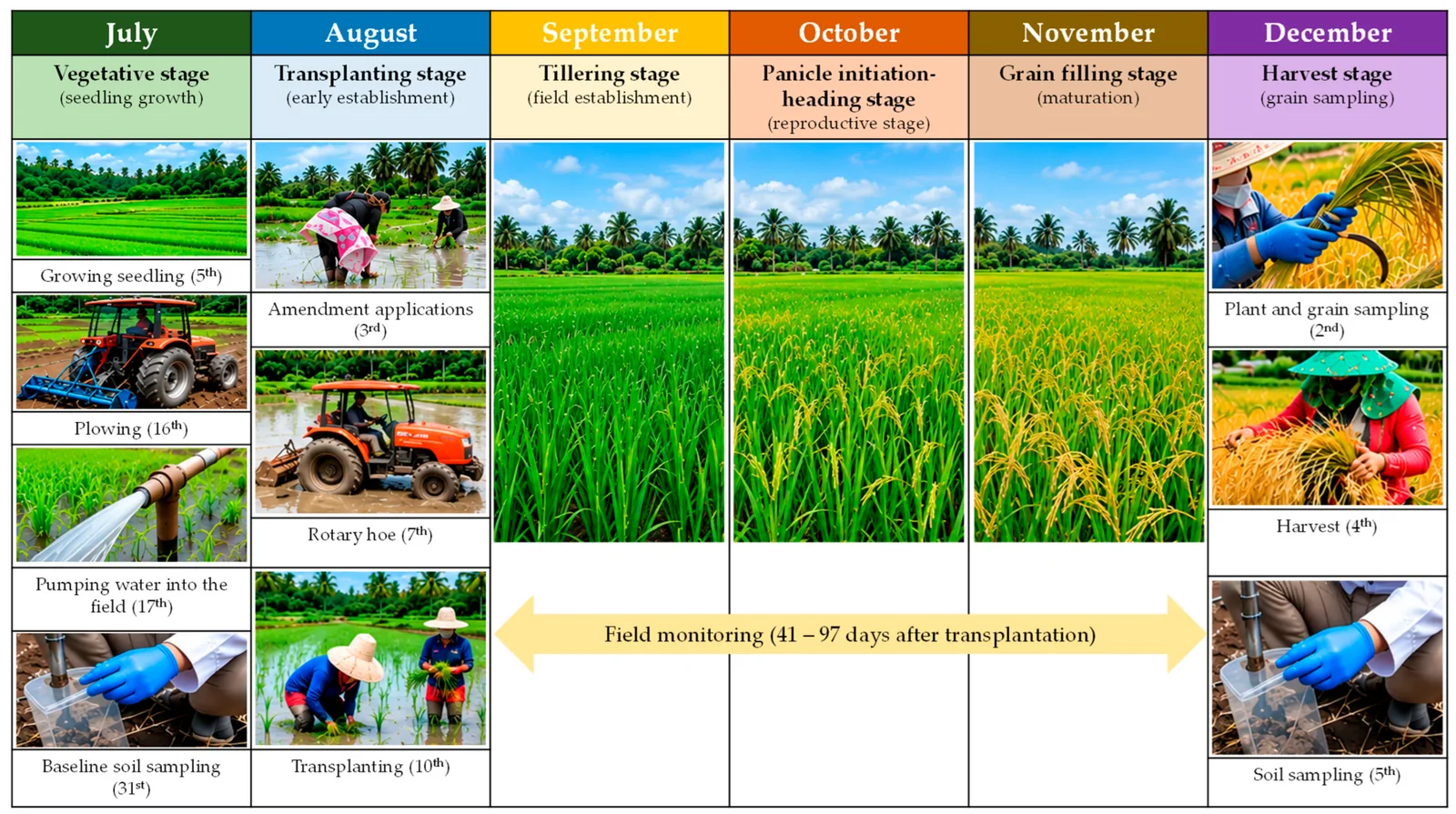
Itinerary of rice experiment in San Pa Tong, Chiang Mai, Thailand.

Rice seedlings were raised on 5 July 2024. The field was plowed on 16 July to incorporate green manure (*Crotalaria juncea* L.) to a depth of approximately 25 cm and was flooded on 17 July to facilitate land preparation for transplanting (Figure 6). Baseline soil samples were collected on 31 July. RHB and KH were applied once on 3 August 2024, after flooding and before rice transplanting, at rates of 15 t/ha and 6.3 kg/ha, respectively. Amendment quantities were calculated according to the prescribed application rates and adjusted to the actual area of each plot. Both amendments were weighed separately for each plot and manually broadcast as uniformly as possible over the soil surface. KH was applied in solid form without dilution or additional carrier water. Cow manure (1.25 t/ha) was uniformly applied to all plots following local farming practice as a common background organic amendment. On 7 August, the soil was rotary-hoed to a depth of approximately 20 cm to incorporate the amendments. Thus, RHB and KH were applied after initial flooding for land preparation and incorporated into the soil before rice transplanting.

Rice seedlings were manually transplanted on 10 August 2024 at a spacing of 35 × 35 cm, with approximately three seedlings per hill, corresponding to a planting density of approximately 8.2 hills/m^2^. Except for the assigned RHB and KH treatments, all plots received identical crop management throughout the experiment.

Water management followed conventional lowland rice cultivation practices in northern Thailand, with irrigation supplied by groundwater pumping and supplemented by natural rainfall. Following transplanting, all plots were maintained under continuous flooding, with a standing water depth of approximately 5–10 cm throughout most of the growing season. Water depth was adjusted according to rainfall and field conditions to maintain comparable flooding conditions among treatments. The plots were drained 10–14 days before harvest to promote grain maturation and facilitate harvesting.

To minimize damage caused by golden apple snails (*Pomacea canaliculata*) and rice-field crabs, a fermented botanical extract prepared from fresh *Senna siamea* leaves was uniformly applied to all plots on 17 and 24 August 2024. No mineral fertilizer was applied during the experiment to avoid introducing an additional readily available nutrient source that could confound the assessment of RHB and KH effects on soil nutrient dynamics and plant nutrient uptake. Because RHB and KH themselves contained nutrients, amendment-derived nutrient inputs were estimated from their application rates and measured nutrient concentrations reported in Table 1; the calculated inputs are presented in Appendix A (Table A2). The nutrient contributions from the incorporated green manure and cow manure could not be quantified because their biomass/application amounts and nutrient compositions were not measured before incorporation. However, both were applied uniformly across the experimental field before treatment establishment and therefore represented common, although unquantified, background nutrient inputs to all treatments. Consequently, their contributions cannot be separated from the overall nutrient supply of the field system, but they do not account for differences in management among the RHB, KH, RHB + KH, and control treatments.

Rice was harvested on 2 December 2024, approximately 114 days after transplanting, and post-harvest soil samples were collected on 5 December 2024.

### 3.3. Chemicals and Reagents

All chemicals and reagents used in this study were of analytical or HPLC grade and were obtained from RCI Labscan (Bangkok, Thailand), unless otherwise specified. Analytical standards were purchased from Sigma-Aldrich (St. Louis, MO, USA), and multi-element calibration standards were supplied by PerkinElmer Inc. (Waltham, MA, USA). 

### 3.4. Soil Sampling and Analyses

Soil samples were collected after harvest. Five soil cores (0–20 cm depth) were randomly collected from each plot using a 10-cm-diameter auger and composited into one representative sample per plot, resulting in 16 composite samples (four replicate samples per treatment). Samples were divided into two portions. One portion was refrigerated at 4 °C and subsequently extracted for NO_3_^−^ and NH_4_^+^ analyses. The second portion was air-dried, sieved (<2 mm), and stored in ziplock bags prior to physicochemical analysis.

#### 3.4.1. Soil pH and Electrical Conductivity

Soil pH and EC were measured in a 1:2 soil-to-deionized water suspension using a multifunction meter (EZ-9909, Sysmatec, Eyholz, Switzerland), following [95].

#### 3.4.2. Soil Macronutrients

Soil TN was determined using the Kjeldahl method [96]. Soil NO_3_^−^ was extracted with 0.5 potassium sulfate (K_2_SO_4_) and determined colorimetrically using the salicylic acid method at 410 nm, following a modified procedure described in [97]. Soil NH_4_^+^ was extracted with 2 M potassium chloride (KCl) and determined colorimetrically using the phenol–nitroprusside method at 636 nm [97].

Available P was extracted using the Olsen method and determined colorimetrically by the molybdenum blue method at 882 nm [35]. Exchangeable K was extracted with 1 N ammonium acetate (NH_4_OAc, pH 7.0) and quantified by atomic absorption spectrophotometry (model 2380, Perkin-Elmer Corporation, Norwalk, CT, USA) at 766.5 nm [98].

### 3.5. Shoot Sampling and Analyses

At rice maturity, shoots and grains were harvested by cutting plants approximately 5 cm above the ground from a 1-m^2^ sampling area within each plot. Rice panicles were separated from the shoots using stainless-steel scissors. Fresh shoot samples were oven-dried at 60 °C to constant weight, and the dry shoot biomass from each 1-m^2^ sampling area was recorded. The dried shoot material was ground using a mortar and pestle and sieved through a 1-mm sieve. Ground shoot samples were acid-digested using nitric acid solution (HNO_3_) and hydrogen peroxide (H_2_O_2_) following U.S. EPA Method 3050B [99]. The concentrations of N, P, and K in rice shoots were subsequently determined using the corresponding analytical procedures described in Section 3.4.2.

Shoot N, P, and K accumulation was calculated on a dry-matter (DM) basis by multiplying the nutrient concentration measured in the dried shoot tissue by the corresponding shoot dry biomass for each plot:

(1) Shoot nutrient accumulation (kg/ha) = C × B × 10

where C is the shoot nutrient concentration (g/kg DM), B is the shoot dry biomass (kg/m^2^), and 10 converts g/m^2^ to kg/ha. Panicles were separated before analysis; therefore, the calculated values represent nutrient accumulation in shoot (straw) biomass only and exclude nutrients contained in the grain.

### 3.6. Grain Harvest and Analyses

Fresh grain weight was recorded immediately after harvest. Grain yield was determined from one 1-m^2^ harvest area located within the interior of each experimental plot and away from plot boundaries and earthen bunds to minimize edge effects. The harvest area was selected to represent the established rice stand within each plot. One 1-m^2^ area was harvested per plot. The harvested paddy rice was then sun-dried under full sunlight for approximately 3 days, following local post-harvest practices, until the grain moisture content was reduced to below 15%. This drying process minimized the risk of grain discoloration and fungal development during storage and milling. Grain yield (t/ha) was calculated by converting the dry grain weight obtained from the 1-m^2^ harvest area to a hectare basis. The dried paddy rice was subsequently dehulled and milled using a rice mill (SUN brand, J.B.J Service Co., Ltd., Chiang Mai, Thailand). The resulting milled rice was weighed and used for subsequent grain quality and nutritional analyses.

#### 3.6.1. Grain Color Measurement

Grain color was evaluated using digital image analysis based on Udomkun et al. [100], with slight modifications. Approximately 30 g of dehulled rice from each plot was evenly distributed on a uniform plate within a controlled illumination chamber and photographed using a digital camera (D3200, Nikon Corp., Tokyo, Japan) positioned 25 cm above the sample. The camera axis was perpendicular to the sample surface, with the illumination arranged at 45° to minimize glare and enhance diffuse reflection. Images were acquired at a shutter speed of 1/160 s and an aperture of f/3.5, with automatic ISO sensitivity and the flash disabled. Five representative images per sample were analyzed using ImageColorPicker (https://imagecolorpicker.com/) [101], and RGB values were converted to CIE L*, a*, and b* color parameters.

#### 3.6.2. Cooking Quality Evaluation

Cooking time was determined according to Karim et al. [102], with minor modifications, by cooking whole rice grains at 90–95 °C until disappearance of the opaque core; the final cooking time was defined as the time required for complete gelatinization plus 2 min. The water uptake ratio, volumetric expansion ratio, and grain elongation ratio were calculated using Equations (2)–(4), respectively. Gel consistency was determined according to Karim et al. [102], and rice was classified as soft (>60 mm), medium (40–60 mm), or hard (<40 mm) based on gel length.

(2) $$\mathrm{Water}\;\mathrm{uptake}\;\mathrm{ratio}=\frac{\mathrm{weight}\;\mathrm{of}\;\mathrm{cooked}\;\mathrm{rice}\;(g)}{\mathrm{weight}\;\mathrm{of}\;\mathrm{uncooked}\;\mathrm{rice}\;(g)}$$

(3) $$\mathrm{Volumetric}\;\mathrm{expansion}\;\mathrm{ratio}=\;\frac{\mathrm{volume}\;\mathrm{of}\;\mathrm{cooked}\;\mathrm{rice}\;(\mathrm{mL})}{\mathrm{volume}\;\mathrm{of}\;\mathrm{uncooked}\;\mathrm{rice}\;(\mathrm{mL})}$$

(4) $$\mathrm{Elongation}\;\mathrm{ratio}=\frac{\mathrm{final}\;\mathrm{grain}\;\mathrm{length}\;\mathrm{after}\;\mathrm{cooking}\;(\mathrm{mm})}{\mathrm{initial}\;\mathrm{grain}\;\mathrm{length}\;\mathrm{before}\;\mathrm{cooking}\;(\mathrm{mm})}$$

#### 3.6.3. Proximate and Nutrient Composition of Rice Grains

Proximate composition was determined according to standard AOAC methods [103]. Moisture, ash, crude fat, and crude protein contents were determined using standard oven-drying, ashing, Soxhlet extraction, and Kjeldahl methods, respectively, with N content additionally verified by combustion analysis. Ash, crude fat, crude protein, and carbohydrate contents are expressed on a DM basis, and total carbohydrate content was calculated by the difference using Equation (5). Amylose content was determined using the iodine-binding colorimetric method [104].

(5) Carbohydrate (%)=100−(Ash+Fat+Protein)

Grain P concentration was determined using the vanadomolybdate colorimetric method following acid digestion [105]. Grain K and Zn concentrations were determined by flame atomic absorption spectrophotometry (Perkin-Elmer AAnalyst 200, Perkin-Elmer Corporation, Norwalk, CT, USA) following Zmozinski et al. [106], with slight modifications, at wavelengths of 766.5 and 213.9 nm, respectively. Element concentrations were calculated using standard calibration curves and are expressed on a dry-weight basis.

#### 3.6.4. Amino Acid Profiling

Amino acid composition was determined according to AOAC Method 994.12, with minor modifications [107]. Grain samples from the four independent experimental plots of each treatment were analyzed separately (*n* = 4 biological replicates). Each sample was injected three times into the HPLC as analytical replicates, and the mean of the three injections was used as the analytical value for each biological replicate. Ground rice samples were oxidized with performic acid and hydrolyzed with 6 M HCl containing phenol at 110 °C for 24 h. Amino acids were derivatized with *o*-phthalaldehyde (OPA) for primary amines and fluorenylmethyloxycarbonyl chloride (FMOC-Cl) for secondary amines and analyzed using an HPLC system (1260 Infinity, Agilent Technologies Inc., Santa Clara, CA, USA) equipped with a ZORBAX Eclipse AAA column (2.1 × 150 mm, 3.5 µm) and a diode array detector. Separation was performed using 40 mM phosphate buffer (pH 7.8) and methanol–acetonitrile–water (45:45:10, *v*/*v*/*v*) as the mobile phases at a flow rate of 0.5 mL/min and a column temperature of 40 °C.

Amino acids were identified by comparing their retention times with those of authentic amino acid standards and quantified using external calibration curves prepared from a commercial amino acid standard mixture. Calibration curves showed good linearity (*R*^2^ > 0.999). Analytical precision was <10% relative standard deviation (RSD), and recoveries from samples fortified at three concentration levels ranged from 80% to 105%. Each biological sample was injected three times as analytical replicates, and the mean of the three injections was used for subsequent analysis. Amino acid contents are expressed as g/100 g protein. The limits of detection (LODs) and quantification (LOQs) were approximately 0.01 and 0.03 µg/mL, respectively. Tryptophan was not quantified because it is degraded during acid hydrolysis. Tyrosine was not considered quantitatively reliable following performic-acid oxidation and was therefore excluded from statistical interpretation. Following acid hydrolysis, asparagine and glutamine are converted to aspartic acid and glutamic acid, respectively; therefore, these values represent the corresponding acid-hydrolysis products.

#### 3.6.5. Total Phenolic Content (TPC) of Rice Grains

Hydrophilic and lipophilic fractions of ground rice grains were sequentially extracted using a modified method [108]. The lipophilic fraction was extracted with tetrahydrofuran, followed by extraction of the hydrophilic fraction from the residue using methanol–acetic acid–water (50:3.7:46.3, *v*/*v*/*v*). The TPC of both fractions was determined using the Folin–Ciocalteu colorimetric method, with absorbance measured at 760 nm using a UV–Vis spectrophotometer (SPECORD^®^ 40, Analytik Jena GmbH, Jena, Germany). TPC was calculated as the sum of the hydrophilic and lipophilic fractions and is expressed as mg gallic acid equivalents (GAE)/100 g DM.

#### 3.6.6. DPPH Antioxidant Activity

The antioxidant activity of the hydrophilic and lipophilic extracts was determined using the DPPH radical-scavenging method [109], with minor modifications. Absorbance was measured at 517 nm using a UV–Vis spectrophotometer, and antioxidant activity was quantified against a Trolox calibration curve and calculated according to Equation (6). Results are expressed as µmol Trolox equivalents (TE)/g DM. Total antioxidant activity was calculated as the sum of the activities measured in the hydrophilic and lipophilic fractions.

(6) $$\mathrm{DPPH}=\frac{{C_{\mathrm{Trolox}\;}V}_{\mathrm{extract}\;}}{m_{\mathrm{sample}}}$$

where *C_Trolox_*_ is the Trolox-equivalent concentration obtained from the calibration curve (µmol/mL), *V_extract_* is the volume of extract used (mL), and *m_sample_* is the dry mass of the sample (g).

### 3.7. Statistical Analysis

All statistical analyses were conducted using R software (version 4.6.0; R Foundation for Statistical Computing, Vienna, Austria) through RStudio (version 2022.02.1; RStudio, PBC, Boston, MA, USA). The experiment followed an RCBD with a 2 × 2 factorial treatment structure comprising RHB and KH. Soil, shoot nutrient accumulation, grain yield and quality, and amino acid variables were analyzed separately, with the individual field plot as the experimental unit (*n* = 4 per treatment; 16 plots in total). For amino acid analysis, the mean of three HPLC injections per plot-level sample was used for statistical analysis.

For each response variable, the main effects of RHB and KH and their interaction were evaluated using the linear model lm(response ~ Block + RHB × KH, data = data). Model assumptions were assessed using residual-versus-fitted and normal Q–Q plots, together with Shapiro–Wilk and Levene’s tests. Given the limited replication, formal tests were interpreted together with graphical residual diagnostics, representative examples of which are provided in Appendix B. Estimated marginal means for the four treatment combinations (control, RHB, KH, and RHB + KH) were compared using Tukey-adjusted pairwise comparisons with the *emmeans* package (version 2.0.4), particularly to characterize treatment differences where RHB × KH interactions were significant. Partial eta-squared (ηp^2^) was calculated as the effect-size measure for each factorial effect. Exact *p*-values and ηp^2^ values were reported for the RHB and KH main effects and their interaction, while lowercase letters indicate significant Tukey-adjusted pairwise differences among treatment combinations (*p* < 0.05).

PCA was conducted using FactoMineR(version 2.17), with biplots generated using factoextra (version 2.2.0). Two datasets were analyzed separately: (i) 27 variables representing soil properties, shoot nutrient accumulation, grain yield, and grain composition and quality, and (ii) 26 variables representing soil properties, grain N and protein contents, and grain amino acid composition. PCA used individual plot-level observations (*n* = 16; four treatments × four replicate plots), with variables centered and scaled to unit variance. Variables were selected according to their relevance to the predefined analytical domains and availability at the plot level, rather than statistical significance or PCA loadings. Given the relatively high variable-to-observation ratio, PCA was interpreted as an exploratory assessment of covariance and multivariate patterns rather than as a confirmatory analysis or evidence of causal relationships.

## 4. Conclusions

This field study showed treatment-associated differences in soil nutrient status, shoot nutrient accumulation, grain yield, and grain composition and quality in flooded rice. RHB primarily increased soil pH, shoot N, P, and K accumulation, and milled rice yield, along with changes in grain nutrient composition and selected quality attributes. KH increased soil N status, shoot N and P accumulation, and both paddy and milled rice yields, as well as grain protein content and selected amino acid concentrations. The combined RHB + KH treatment also affected soil N status, shoot nutrient accumulation, and paddy rice yield, together with several grain nutritional and quality attributes. Factorial analysis further showed that several responses depended on RHB × KH interactions, particularly for soil nutrient properties, grain composition, and amino acid profiles, indicating that the effects of co-application were not consistently additive. Overall, grain composition and quality responses were parameter-specific rather than uniformly favorable across all measured attributes.

The concurrent responses observed across soil, shoot, and grain measurements indicate that RHB and KH affected multiple components of the production system but do not establish causal nutrient-transfer pathways among these components. Consideration of amendment-derived nutrient inputs further indicates that direct nutrient addition may have contributed to responses under RHB, whereas the comparatively small nutrient inputs from KH suggest that its effects, particularly on shoot N accumulation, cannot be explained by direct nutrient supply alone. PCA identified multivariate associations among soil properties, shoot nutrient accumulation, grain nutritional composition, yield, and quality traits, but these associations should be interpreted as exploratory rather than evidence of nutrient-transfer mechanisms. Collectively, the findings demonstrate distinct and amendment-specific effects of RHB and KH and show that their combined application did not consistently produce greater responses than either amendment alone.

As this field experiment was conducted at a single site over one rice-growing season, the consistency of the observed responses across years, locations, and contrasting environmental conditions remains to be established. The findings should therefore be interpreted within the environmental and management conditions of the present study. In addition, the relative contributions of direct amendment nutrient inputs, changes in soil nutrient availability, and plant nutrient acquisition could not be fully resolved from the measurements available. The absence of a conventional mineral-fertilizer or farmer-practice benchmark also limits direct comparison of the agronomic performance of RHB and KH with standard fertilization practices. Future multi-year and multi-site field studies incorporating conventional fertilizer benchmarks, seasonal nutrient fluxes, soil nutrient fractions, plant biomass and nutrient allocation, and complete nutrient-input and output balances would help clarify the processes underlying RHB and KH responses and determine their stability under varying soil, climatic, and management conditions.

## Figures and Tables

**Table 2:** Effects of rice husk biochar (RHB), potassium humate (KH), and their combination on rice grain characteristics, proximate composition, nutritional composition, bioactive compounds, and amino acid profiles relative to the control.

Rice Grain Characteristics	Control	RHB	KH	RHB × KH
Visual color	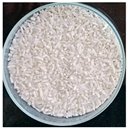	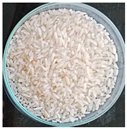	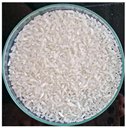	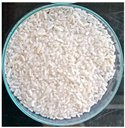
Lightness (L*, –)	89.2 (0.3) c	91.3 (0.2) b	90.2 (0.3) b	98.1 (0.2) a
Redness (a*, –)	1.32 (0.01) b	2.35 (0.01) a	2.14 (0.01) a	1.23 (0.01) b
Yellowness (b*, –)	3.22 (0.01) b	5.23 (0.01) a	2.38 (0.01) c	3.15 (0.01) b
Cooking time (min)	24 (3) a	26 (1) a	25 (3) a	26 (2) a
Water uptake ratio (–)	3.4 (0.1) a	3.8 (0.6) a	3.9 (1.0) a	3.4 (0.2) a
Volumetric expansion ratio (–)	2.4 (0.1) a	3.4 (0.4) a	2.9 (0.1) a	3.0 (0.1) a
Elongation ratio (–)	1.4 (0.1) b	1.4 (0.1) b	1.48 (0.01) a	1.3 (0.1) b
Gel consistency (mm)	<40 a	<40 a	<40 a	<40 a
Proximate and nutritional composition of rice grain (% DM, except moisture which is on fresh weight basis)				
Carbohydrate	89.2 (0.1) a	88.7 (0.1) b	87.9(0.1) c	88.3 (0.1) c
Protein	9.9 (0.1) d	10.2 (0.1) c	10.96 (0.01) a	10.6 (0.1) b
Fat	0.38 (0.01) c	0.55 (0.03) a	0.53 (0.01) a	0.48 (0.01) b
Ash	0.51 (0.01) c	0.70 (0.03) a	0.56 (0.01) b	0.60 (0.01) b
Moisture content	13.6 (0.1) c	13.22 (0.03) d	13.81 (0.02) b	14.13 (0.02) a
Nitrogen	1.66 (0.01) d	1.71 (0.01) c	1.84 (0.01) a	1.78 (0.01) b
Phosphorus	0.10 (0.01) d	0.14 (0.01) a	0.12 (0.01) c	0.13 (0.01) b
Potassium	0.061 (0.001) d	0.105 (0.004) b	0.087 (0.002) c	0.128 (0.004) a
Zinc (Zn, mg/100 g DM)	2.4 (0.1) a	2.48 (0.03) a	2.13 (0.03) c	2.3 (0.1) b
Amylose	18.5 (0.5) a	17.7 (0.2) b	17.6 (0.4) b	17.2 (0.3) b
Bioactive compound				
DPPH antioxidant activity (µmol Trolox eq/g DM)	0.46 (0.07) a	0.62 (0.09) a	0.30 (0.06) b	0.54 (0.06) a
Total phenolic content (mg GAE/100 g DM)	9.62 (0.20) a	8.43 (0.09) c	7.62 (0.15) d	8.94 (0.20) b
Amino acid composition (g/100 g)				
Alanine	1.22 (0.01) b	1.48 (0.01) ab	1.37 (0.01) b	1.68 (0.02) a
Arginine	1.40 (0.01) b	1.78 (0.01) a	1.64 (0.02) ab	1.87 (0.01) a
Aspartic acid	1.68 (0.01) c	2.08 (0.02) b	2.19 (0.01) b	2.33 (0.01) a
L-cystine	0.198 (0.001) a	0.198 (0.010) a	0.198 (0.010) a	0.198 (0.010) a
Glutamic acid	3.56 (0.02) c	3.66 (0.02) c	4.75 (0.03) b	5.04 (0.03) a
Glycine	1.58 (0.02) d	1.88 (0.01) c	1.98 (0.02) b	2.08 (0.02) a
Histidine	0.10 (0.00) b	0.40 (0.01) a	0.50 (0.01) a	0.40 (0.01) a
Isoleucine	1.38 (0.02) b	0.20 (0.01) c	0.30 (0.01) c	1.88 (0.03) a
Leucine	0.79 (0.02) c	1.68 (0.01) a	1.78 (0.02) a	1.09 (0.01) b
Lysine	0.10 (0.01) b	0.89 (0.02) a	0.99 (0.02) a	0.10 (0.01) b
Methionine	0.69 (0.01) b	0.10 (0.01) c	0.20 (0.01) c	1.09 (0.02) a
Phenylalanine	1.09 (0.02) b	0.99 (0.01) b	1.09 (0.01) b	1.68 (0.02) a
Proline	1.09 (0.01) c	1.78 (0.02) a	1.58 (0.02) b	1.48 (0.02) b
Serine	0.49 (0.01) d	1.19 (0.01) b	1.38 (0.01) a	0.69 (0.01) c
Threonine	1.38 (0.01) b	0.59 (0.01) c	0.49 (0.01) c	1.88 (0.02) a
Valine	0.10 (0.01) b	0.30 (0.01) a	0.30 (0.01) a	0.30 (0.01) a

Values in parentheses are standard errors. ND is not detected. Lowercase letters (a–d) denote significant differences among treatments as determined by Tukey’s HSD test (*p* < 0.05).

**Figure A1:**
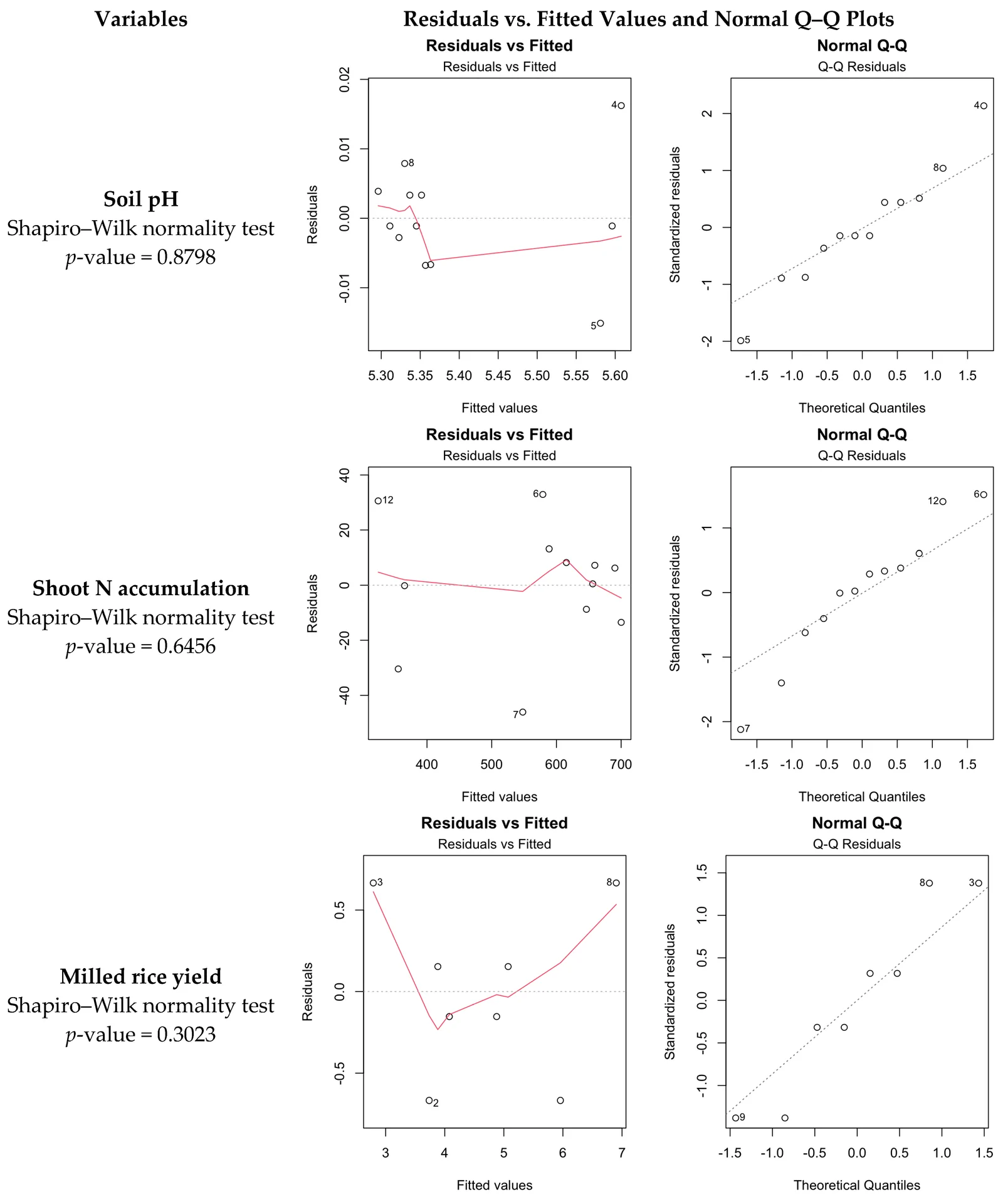

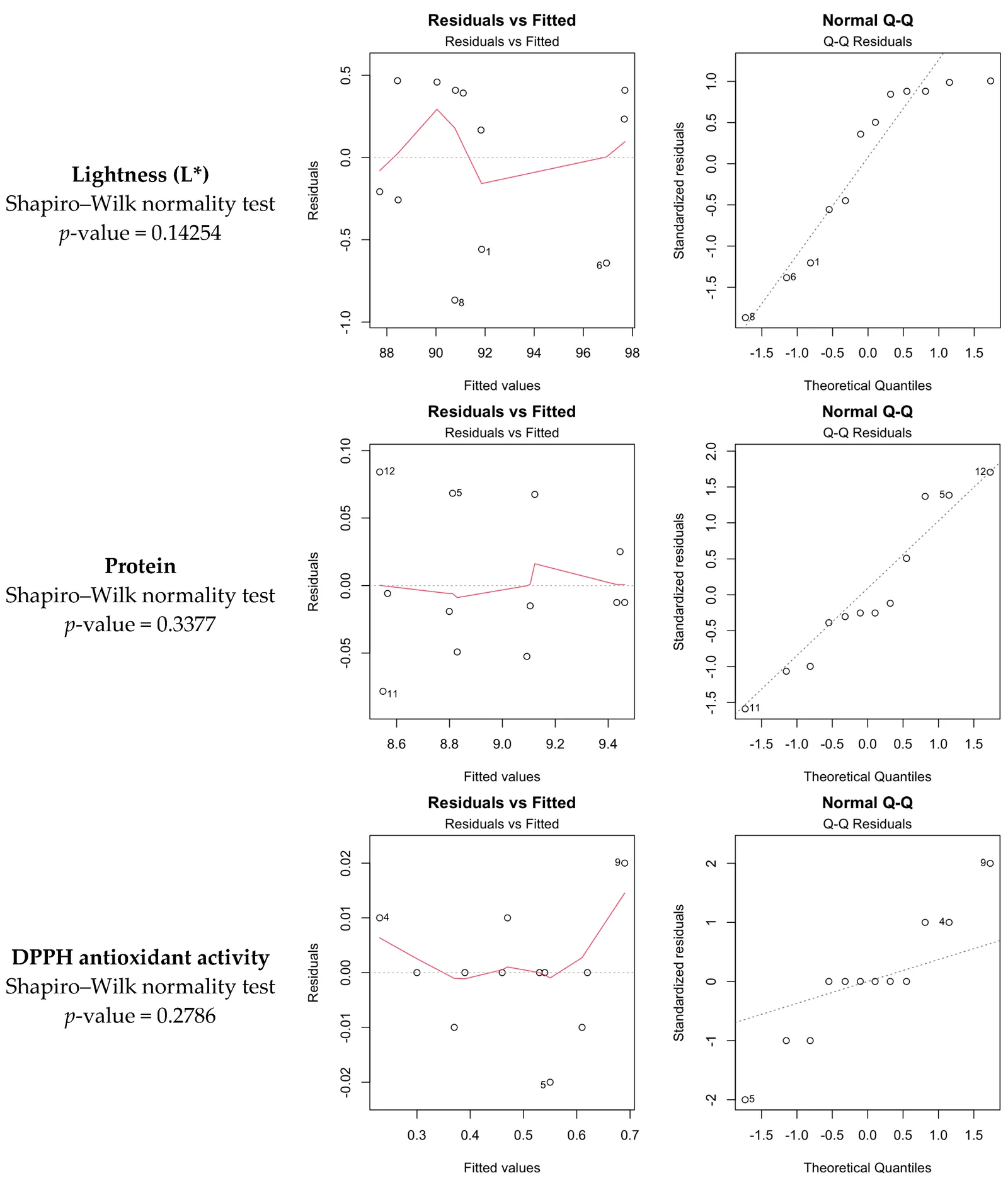

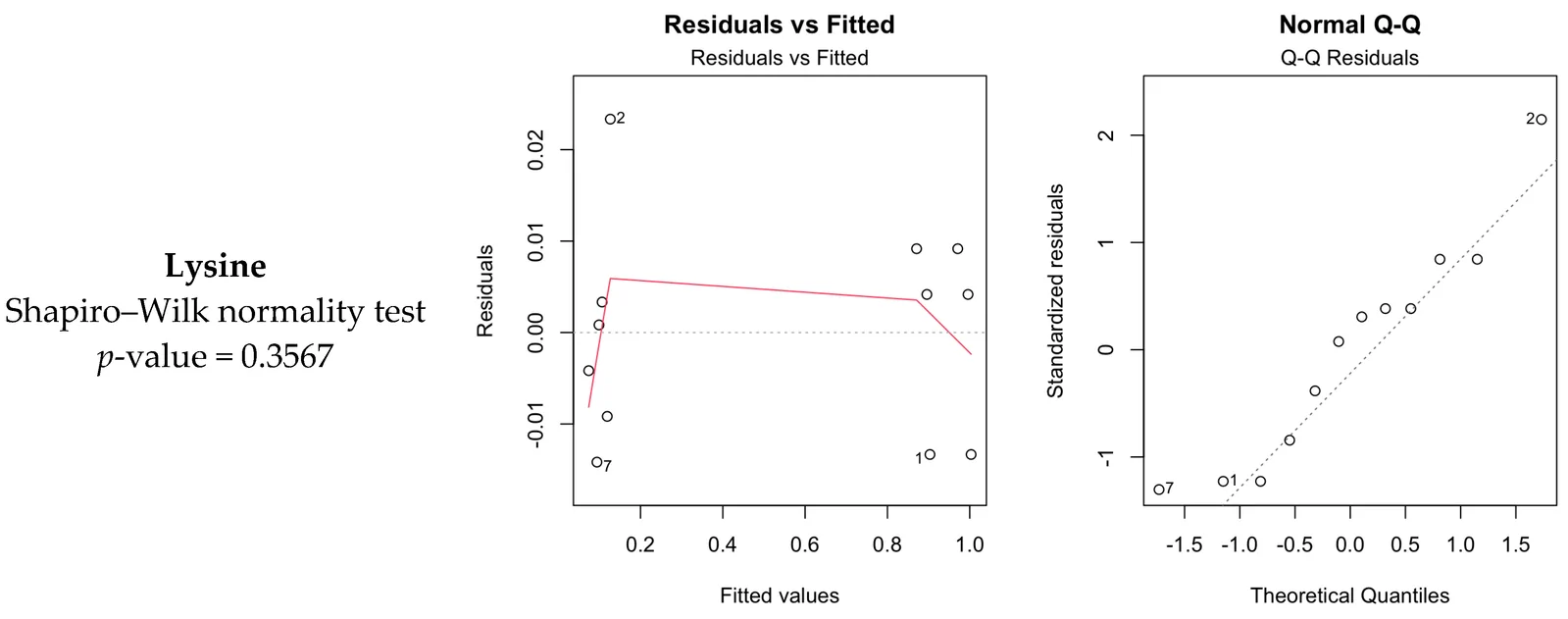
Representative residual diagnostic plots for the factorial ANOVA models. Residual-versus-fitted and normal Q–Q plots are shown for selected variables representing soil properties, shoot nutrient accumulation, yield, grain composition and quality, and amino acid composition.

## Data Availability

The datasets used and/or analyzed during the current study are available from the corresponding authors upon reasonable request.

## Abbreviations

The following abbreviations are used in this manuscript:

AAS	Atomic Absorption Spectrophotometer (or Atomic Absorption Spectroscopy, depending on context)
AOAC	Association of Official Analytical Chemists
CEC	Cation Exchange Capacity
CIE	Commission Internationale de l’Éclairage
DAD	Diode Array Detector
DI	Deionized
DM	Dry Matter
DNRA	Dissimilatory Nitrate Reduction to Ammonium
DPPH	1,1-Diphenyl-2-picrylhydrazyl
EC	Electrical Conductivity
EDTA	Ethylenediaminetetraacetic Acid
FMOC-Cl	Fluorenylmethyloxycarbonyl Chloride
GAE	Gallic Acid Equivalents
HPLC	High-Performance Liquid Chromatography
HSD	Honestly Significant Difference
KDML105	Khao Dawk Mali 105
KH	Potassium Humate
LOD	Limit of Detection
LOQ	Limit of Quantification
NH_3_	Ammonia
NH_4_^+^	Ammonium
NO_3_^−^	Nitrate
OM	Organic Matter
OPA	*o*-Phthalaldehyde
PCA	Principal Component Analysis
RCBD	Randomized Complete Block Design
RHB	Rice Husk Biochar
RHB + KH	Combined application of Rice Husk Biochar and Potassium Humate
RGB	Red–Green–Blue
SOC	Soil Organic Carbon
TN	Total Nitrogen
TPC	Total Phenolic Content
UV–Vis	Ultraviolet–Visible (Spectrophotometry)

## Appendix A

### Appendix A.1. Factorial ANOVA Results for RHB, KH, and Their Interaction

Factorial ANOVA was used to evaluate the main effects of RHB, KH, and their interaction (RHB × KH) on soil properties, shoot nutrient accumulation, grain yield, and grain composition and quality. RHB and KH were treated as fixed factors, with block included to account for the randomized complete block design.

**Table A1:** Factorial ANOVA results for the effects of rice husk biochar (RHB), potassium humate (KH), and their interaction (RHB × KH) on soil, plant, yield, and grain properties.

Property	RHB			KH			RHB × KH		
	F-Value	*p*-Value	ηp^2^	F-Value	*p*-Value	ηp^2^	F-Value	*p*-Value	ηp^2^
Soil properties									
Soil pH	28.76	<0.001	0.762	55.21	<0.001	0.860	50.35	<0.001	0.848
Soil EC	51.16	<0.001	0.850	67.24	<0.001	0.882	62.52	<0.001	0.874
Total N	28.03	<0.001	0.757	71.05	<0.001	0.888	3.85	0.081	0.300
NO_3_^−^–N	156.40	<0.001	0.946	1.75	0.219	0.163	157.39	<0.001	0.946
NH_4_^+^–N	130.50	<0.001	0.935	48.52	<0.001	0.844	139.19	<0.001	0.939
Available P	138.05	<0.001	0.939	59.79	<0.001	0.869	125.02	<0.001	0.933
Exchangeable K	86.82	<0.001	0.906	110.65	<0.001	0.925	0.85	0.381	0.086
Shoot nutrient accumulation									
N accumulation	56.84	<0.001	0.863	25.58	<0.001	0.740	128.55	<0.001	0.935
P accumulation	10.42	0.010	0.537	38.44	<0.001	0.810	9.59	0.013	0.516
K accumulation	155.61	<0.001	0.945	0.56	0.473	0.059	0.28	0.610	0.030
Yield									
Paddy grain yield	2.21	0.171	0.197	12.35	0.007	0.578	4.54	0.062	0.335
Milled grain yield	2.00	0.191	0.182	9.27	0.014	0.507	2.52	0.147	0.219
Grain physical/cooking properties									
Lightness (L*)	184.92	<0.001	0.954	116.25	<0.001	0.928	21.35	0.001	0.703
Redness (a*)	2.43	0.153	0.213	15.19	0.004	0.628	25.21	<0.001	0.737
Yellowness (b*)	130.44	<0.001	0.935	42.44	<0.001	0.825	26.12	<0.001	0.744
Cooking time	3.90	0.080	0.302	0.62	0.451	0.064	1.40	0.267	0.135
Water uptake ratio	0.04	0.846	0.004	0.06	0.812	0.007	1.48	0.255	0.141
Volumetric expansion ratio	5.34	0.046	0.372	0.07	0.797	0.008	3.25	0.105	0.265
Elongation ratio	3.52	0.093	0.281	0.12	0.737	0.013	4.67	0.059	0.342
Gel consistency	1.00	0.343	0.100	1.00	0.343	0.100	1.00	0.343	0.100
Proximate/nutritional composition									
Carbohydrate	37.63	<0.001	0.807	64.17	<0.001	0.877	71.51	<0.001	0.888
Protein	0.91	0.365	0.092	218.36	<0.001	0.960	56.13	<0.001	0.862
Fat	10.36	0.011	0.535	4.59	0.061	0.338	37.30	<0.001	0.806
Ash	32.19	<0.001	0.782	1.37	0.272	0.132	14.92	0.004	0.624
Moisture content	0.86	0.378	0.087	113.67	<0.001	0.927	104.13	<0.001	0.920
Grain N	0.90	0.368	0.091	212.64	<0.001	0.959	55.48	<0.001	0.860
Grain P	353.27	<0.001	0.975	81.72	<0.001	0.901	3.82	0.082	0.298
Grain K	607.23	<0.001	0.985	186.94	<0.001	0.954	1.72	0.222	0.160
Zn	23.05	<0.001	0.719	44.18	<0.001	0.831	1.23	0.296	0.120
Amylose	28.47	<0.001	0.760	55.97	<0.001	0.861	2.05	0.186	0.186
Bioactive compounds									
DPPH antioxidant activity	60.00	<0.001	0.870	21.60	0.001	0.706	24.00	<0.001	0.727
Total phenolic content (TPC)	0.33	0.580	0.035	41.98	<0.001	0.823	97.19	<0.001	0.915
Amino-acid profile									
Alanine	26.27	<0.001	0.745	10.21	0.011	0.531	2.33	0.161	0.206
Arginine	20.29	0.001	0.693	12.14	0.007	0.574	2.45	0.152	0.214
Aspartic acid	28.48	<0.001	0.760	5.66	0.041	0.386	7.04	0.026	0.439
L-cystine	0.02	0.891	0.002	0.50	0.497	0.053	0.22	0.650	0.024
Glutamic acid	9.00	0.015	0.500	38.37	<0.001	0.810	20.65	0.001	0.696
Glycine	11.26	0.008	0.556	25.20	<0.001	0.737	2.82	0.127	0.239
Histidine	7.10	0.026	0.441	18.52	0.002	0.673	18.01	0.002	0.667
Isoleucine	5.73	0.040	0.389	1.22	0.298	0.119	23.80	<0.001	0.726
Leucine	1.16	0.309	0.114	4.65	0.059	0.341	72.48	<0.001	0.890
Lysine	2.03	0.188	0.184	4.57	0.061	0.337	88.94	<0.001	0.908
Methionine	2.28	0.165	0.202	6.06	0.036	0.402	50.37	<0.001	0.848
Phenylalanine	14.80	0.004	0.622	29.12	<0.001	0.764	22.15	0.001	0.711
Proline	9.72	0.012	0.519	1.15	0.311	0.113	17.28	0.002	0.658
Serine	0.15	0.708	0.016	4.98	0.053	0.356	65.96	<0.001	0.880
Threonine	7.16	0.025	0.443	3.08	0.113	0.255	86.88	<0.001	0.906

Note: RHB, rice husk biochar; KH, potassium humate. RHB, KH, and RHB × KH each had 1 numerator degree of freedom, with 9 residual degrees of freedom. Partial eta-squared (ηp^2^) was calculated as an effect-size measure for each factorial effect as ηp^2^ = (F × df_effect)/(F × df_effect + df_error). Exact *p*-values are reported to three decimal places when *p* ≥ 0.001; values below 0.001 are reported as *p* < 0.001. Statistical significance was determined at *p* < 0.05.

### Appendix A.2. Estimated Nutrient Inputs from RHB and KH

Nutrient inputs associated with RHB and KH were estimated from their application rates and measured nutrient concentrations reported in Table 1. The calculated values are provided in Table A1.

**Table A2:** Estimated nutrient inputs from RHB and KH based on amendment application rates and measured nutrient concentrations reported in Table 1. RHB and KH were applied at 15 t/ha and 6.3 kg/ha, respectively.

Treatment	Total N Input (kg/ha)	Available P Input (kg/ha)	Available K Input (kg/ha)
Control	0.00	0.00	0.00
RHB	65.26	1.11	39.16
KH	0.022	0.0005	0.046
RHB + KH	65.28	1.11	39.21

Note: Nutrient inputs were estimated from the amendment application rates and measured nutrient concentrations reported in Table 1. N values represent total N inputs, whereas P and K values were calculated from measured available P and available K concentrations and therefore do not represent total P and K additions. The nutrient contribution from incorporated green manure (*Crotalaria juncea* L.) could not be quantified because its biomass and nutrient composition were not measured before incorporation. Cow manure was applied at 1.25 t/ha, but its nutrient composition was not determined; therefore, its N, P, and K inputs could not be quantified. Green manure and cow manure were applied uniformly across the experimental field before treatment establishment and therefore represented common, but unquantified, background nutrient inputs to all treatments.

## Appendix B

### Residual Diagnostic Plots for Factorial ANOVA Models

Residual diagnostics were used to assess the assumptions of the factorial ANOVA models. Residuals-versus-fitted and normal Q–Q plots were examined for all response variables, together with Shapiro–Wilk tests of residual normality. Representative diagnostic plots for the major response categories are presented below. Given the limited replication, formal normality tests were interpreted together with graphical diagnostics. For the representative models shown, no substantial departures from normality were evident, and all Shapiro–Wilk tests were non-significant (*p* > 0.05).
